# The Impact of Dietary Interventions on Metabolic Outcomes in Metabolic Dysfunction-Associated Steatotic Liver Disease (MASLD) and Comorbid Conditions, Including Obesity and Type 2 Diabetes

**DOI:** 10.3390/nu17071257

**Published:** 2025-04-03

**Authors:** Joanna Michalina Jurek, Katarzyna Zablocka-Sowinska, Helena Clavero Mestres, Leyre Reyes Gutiérrez, Javier Camaron, Teresa Auguet

**Affiliations:** 1 Grup de Recerca GEMMAIR (AGAUR)—Medicina Aplicada (URV), Departament de Medicina i Cirurgia, Institut d’Investigació Sanitària Pere Virgili (IISPV), Universitat Rovira i Virgili (URV), Mallafré Guasch, 4, 43007 Tarragona, Spain; joannamichalina.jurek@urv.cat (J.M.J.); helena.clavero@urv.cat (H.C.M.); leyre.reyes@estudiants.urv.cat (L.R.G.); 2 The Faculty of Finance and Management, WSB Merito University in Wrocław, 53-609 Wrocław, Poland; katarzyna.zablocka-slowinska@wroclaw.merito.pl; 3 Servei Medicina Interna, Hospital Universitari de Tarragona Joan XXIII, Mallafré Guasch, 4, 43007 Tarragona, Spain; jcamaronm.hj23.ics@gencat.cat

**Keywords:** dietary intervention, metabolic outcomes, obesity, MASLD/non-alcoholic fatty liver disease, type 2 diabetes, metabolic syndrome, Mediterranean, systematic review

## Abstract

**Background:** Metabolic dysfunction-associated steatotic liver disease (MASLD) is a public health concern, linked with immune-metabolic dysfunction. While lifestyle and dietary modifications remain the cornerstone of MASLD management, the optimal dietary approach remains uncertain. **Objectives:** This systematic review aims to investigate the impact of model dietary patterns on metabolic outcomes in patients with MASLD and evaluate their effects in individuals with coexisting metabolic conditions, such as obesity, metabolic syndrome, and type 2 diabetes mellitus (T2DM). **Methods:** To conduct the review, PubMed, Scopus, Google Scholar, Cochrane CENTRAL, and ClinicalTrials.gov databases were searched for Randomized Controlled Trials (RCTs) on the adult population, published between January 2019 and September 2024, following PRISMA principles. The quality of the included RCTs was assessed qualitatively based on study characteristics. **Results:** The main findings of this review demonstrated that the use of interventions with dietary model based on Mediterranean diet (MED) and intermittent fasting (IF) approaches, such as alternative-day fasting (ADF) and time-restricted feeding regimens (TRF) may have potential in reducing body weight, BMI, and waist circumference, with additional benefits of improving glycemic control and reducing inflammation. The effects on hepatic functions, although limited, may be linked with reduced enzyme activity and liver stiffness. Additionally, the use of lacto-ovo-vegetarian diet (LOV-D) and the Dietary Approaches to Stop Hypertension (DASH) diet may offer additional health benefits, including blood pressure management. **Conclusions:** This review suggests that MED and IF-based strategies may reduce BW, improve glycemic control, and lower inflammation, with potential benefits for hepatic function. Further long-term studies are needed to confirm these effects and underlying mechanisms, which will allow for the optimization of protocols and ensure their safety in MASLD.

## 1. Introduction

Metabolic dysfunction-associated steatotic liver disease (MASLD) is currently the most prevalent chronic liver disease worldwide, affecting approximately 30% of the global population [1]. The condition, previously known as non-alcoholic fatty liver disease (NAFLD), is driven by an excessive accumulation of fat in more than 5% of hepatocytes and encompasses a spectrum of liver pathologies ranging from simple steatosis to metabolic dysfunction-associated steatohepatitis (MASH), fibrosis, cirrhosis, and hepatocellular carcinoma [2]. The diagnosis of MASLD is based on the presence of hepatic steatosis, confirmed by imaging or histology, in combination with at least one cardiometabolic risk factor, such as obesity, dyslipidemia, insulin resistance, or type 2 diabetes mellitus (T2DM), and the avoidance of excessive alcohol consumption [2,3].

Liver biopsy remains the gold standard for distinguishing between MASLD and MASH, particularly in cases where non-invasive tests yield inconclusive results. Histological assessment identifies steatosis, hepatocellular ballooning, lobular inflammation, and fibrosis, which are key hallmarks of MASH [2]. The key clinical parameters assessed in the MASLD include anthropometric measures (body mass index (BMI), waist circumference (WC)), glucose metabolism (fasting glucose, glycated hemoglobin A1c (HbA1c), insulin resistance indices), lipid profile (triglycerides, high-density lipoprotein cholesterol (HDL), low-density lipoprotein cholesterol (LDL)), and inflammatory markers (C-reactive protein (CRP), tumor necrosis factor alpha (TNF-α), and interleukins (ILs)) [3]. Given the rising prevalence and strong association with cardiometabolic diseases, MASLD represents a significant global health burden, requiring early detection and multidisciplinary management strategies.

Although the use of Resmetirom has recently been approved for patients with noncirrhotic MASH and moderate to advanced fibrosis in the USA [4], lifestyle and dietary interventions play a pivotal role in MASLD management, with various dietary patterns demonstrating beneficial effects on hepatic steatosis, insulin resistance, and inflammation [5].

In this review, we focus on the most widely studied and clinically relevant dietary models: the Mediterranean diet (MED), the Dietary Approaches to Stop Hypertension (DASH) diet, low-fat diets (LFD), low-carbohydrate diets (LCD), the ketogenic diet (KD), and intermittent fasting (IF). These models were selected based on their frequent application in clinical trials and observational studies targeting obesity, hepatic fat accumulation, carbohydrate and lipid profiles, as well as inflammation—key components in the pathogenesis of MASLD. Moreover, these dietary patterns are often recommended in clinical guidelines for managing metabolic conditions, making them highly relevant in the context of MASLD [3,5].

Among the most effective dietary models, the Mediterranean diet (MED) has been extensively studied, showing improvements in hepatic fat content, fibrosis, liver enzymes, glucose metabolism, and lipid profile, supporting its role as a primary intervention for MASLD. The MED emphasizes high consumption of fruits, vegetables, whole grains, nuts, and olive oil as the primary source of fat, with moderate intake of fish and poultry, and minimal consumption of red meat and processed foods. This dietary pattern has been associated with improving hepatic and metabolic profiles due to its high content of monounsaturated fats, polyphenols, and dietary fiber [6,7].

The Dietary Approaches to Stop Hypertension (DASH) diet, originally designed to lower blood pressure and improve cardiovascular health, has also been linked to reduced liver steatosis, insulin resistance, and inflammation, as well as potentially lowering hepatic fat accumulation in MASLD patients. The DASH diet is rich in whole grains, lean proteins, and vegetables, featuring high intakes of fiber, lean proteins, and unsaturated fats while limiting sodium, red meats, and added sugars. This diet has shown promise in improving metabolic and hepatic parameters [8].

Low-fat (LFD) or low-carbohydrate (LCD) diets offer alternative strategies, with evidence suggesting that reducing carbohydrate intake can lower intrahepatic lipid accumulation, triglyceridemia, lipid oxidation, and insulin resistance [9].

Other dietary approaches, such as the ketogenic diet (KD) and intermittent fasting (IF), have gained attention for their potential to improve metabolic state and reduce hepatic fat content, though long-term safety data are still lacking [5]. Although there are different IF approaches, daily time-restricted feeding regimen (TRF) with an 18-h fasting period and a 6-h eating window (16/8) and alternate-day fasting (ADF), characterized by 24-h of fasting at 25% of baseline energy, have recently gained attention as potential interventions in improving the management of metabolic conditions [10].

The Ketogenic Diet (KD), characterized by high fat, moderate protein, and very low carbohydrate intake, aims to induce ketosis, a metabolic state in which fat is used as the primary energy source. Some studies suggest that the KD can lead to weight loss, reduced insulin resistance, and decreased liver fat content [11].

Despite the promising results of various dietary interventions, significant knowledge gaps remain, particularly regarding the optimal macronutrient composition, individual variability in diet response, and long-term adherence to specific dietary patterns [3]. The optimal macronutrient composition (carbohydrates, fats, proteins) for MASLD management remains unclear, particularly when comparing LCD and LFD. Although the Mediterranean diet is well-studied in MASLD, the effectiveness and long-term adherence of other dietary patterns, such as DASH or KD, need further exploration, also in comparison with MED. Additionally, most studies are conducted over a few months, creating a gap in knowledge regarding the long-term effects of specific dietary models on MASLD.

There remains a lack of in-depth discussion regarding the heterogeneity and methodological limitations of existing studies. Many trials differ in duration, sample size, participant characteristics, and adherence monitoring, making direct comparisons between dietary models difficult. Furthermore, unresolved questions persist regarding the mechanisms underlying the differential effects of various diets, especially in populations with comorbidities. Few studies incorporate comprehensive metabolic phenotyping or explore epigenetic and microbiome-mediated responses to diet, which may account for the observed individual variability [8,9].

Given identified gaps, along with a need for a comprehensive comparison of different dietary models, an our hypothesis that MED will be the most effective, with IF offering comparable benefits, the main aim of this review is to provide an updated overview of the benefits of these interventions on metabolic outcomes in MASLD, while highlighting the new research opportunity for long-term, multicenter trials to optimize dietary recommendations for MASLD management accompanied by comorbid conditions, such as obesity and T2DM. 

## 2. Materials and Methods

This review was structured based on the PRISMA (Preferred Reporting Items for Systemic Reviews and Meta-Analyses) guidelines for systematic reviews [12]. The protocol for this study was registered in the PROSPERO (http://www.crd.york.ac.uk/PROSPERO, accessed on 20 August 2024) with registration number CRD42024581563.

The systematic review question was framed using a framework involving patients, intervention, comparison, and outcomes (PICOS), and was defined to assess how nutritional interventions with applied model dietary patterns can impact metabolic outcomes in patients with MASLD alone, and with other metabolic conditions, including obesity, metabolic syndrome, and type 2 diabetes.

### 2.1. Search Strategy

An electronic search through the PubMed, Web of Science, Scopus, and Google Scholar databases to identify articles published up to September 2024, using following search terms for their presence in the study title and/or in the abstract: “diet”, “NAFLD”, “Non-alcoholic Fatty Liver Disease”, “MASLD”, “Metabolic dysfunction-Associated Steatotic Liver Disease”, “obesity”, “type 2 diabetes”, “diabetes mellitus”, “metabolic syndrome”. All searches were adapted to each used database accordingly, and were restricted to studies in English, Polish, or Spanish; date limitations of the period between January 2019 and September 2024 (inclusive) were applied.

### 2.2. Study Selection

The retrieved studies were collected by four researchers on the basis of their titles and abstracts. Then, the full text of all these articles was carefully evaluated by two independent reviewers to extract data on the model dietary patterns, as well as pre-defined metabolic outcomes based on the diagnostic criteria for MASLD [1]. Both of these processes were performed by the first two authors separately, and any disagreements were discussed and resolved with the third researcher.

### 2.3. Selection Criteria

This systematic review included Randomized Controlled Trials (RCTs) published between January 2019 and September 2024, which involved adult individuals, aged between 18 and 65 years of age, who were diagnosed with MASLD alone or with additional comorbidity risk factors, such as obesity, MS, T2DM, or insulin resistance. The interventions included various dietary models such as the MED, IF protocols, LCD, or combinations of these approaches, which were compared to control groups, including those receiving no intervention or standard care, which was defined for each study. The outcomes assessed in this review were the reported baseline and post-intervention values of metabolic outcomes relevant for MASLD diagnosis [1]. 

Studies were excluded if they (1) examined only nutrients; (2) examined single foods or food groups; (3) examined supplements and/or medications rather than a model dietary pattern; (4) were conducted on patients younger than 18 years of age or older than 65 years of age, as well as if the study included pregnant women. Moreover, studies combining (5) dietary interventions with lifestyle changes, including behavioral interventions, cognitive-behavioral therapy, psycho-educational programs, self-management skills, motivational interviewing, and lifestyle changes (physical activity, sleep, stress management, diet quality, and habits) were also excluded.

### 2.4. Data Extraction

Data extracted from each identified study were reported in an Excel file and included the following variables: DOI, study title, first author’s family name, publication year, study design, geographical region where the study was conducted, sample size (separately for each intervention/control), participant details (condition and co-existing comorbidity, mean age, mean BMI and weight, gender), dietary intervention (model diet, intervention duration, type of control/comparison), baseline and post-intervention measures of metabolic outcomes, including:

(1) Anthropometric measures of weight, BMI, WC, body fat percentage (BF), diastolic (DBP), and systolic blood pressure (SBP);

(2) Glucose, lipid metabolism and inflammation biomarkers (fasting glucose, fasting insulin, insulin resistance index (HOMA-IR), glycated hemoglobin (HbA1c), triglycerides, cholesterol, low-density lipoprotein cholesterol (LDL-C), high-density lipoprotein cholesterol (HDL-C), high-sensitivity C-reactive protein (hs-CRP), monocyte chemoattractant protein-1 (MCP-1), and lipopolysaccharides (LPS));

(3) Measures of liver function (aspartate aminotransferase (AST), alanine aminotransferase (ALT), percentage of intrahepatic fat (IHL), controlled attenuation parameter (CAP), liver stiffness (LSM), acoustic radiation force electrography (ARF), fatty liver index (FLI), hepatic steatosis index (HIS), Hepascore, Fibrosis-4 score (FIB-4)).

If available, secondary measures involving determinants of physical activity, smoking status, alcohol intake, sleep and diet quality, stress levels, mental health status, and self-reported quality of life were reported.

In addition, the applied nutritional interventions and used model dietary patterns were described separately, and they are presented in Table 1. The description of each model of dietary pattern included the nutritional composition (percentage of carbohydrate, protein, and fat) along with a brief description of included food groups, meal timing, and number of meals per day, and caloric value.

### 2.5. Risk of Bias

In this review, the risk of bias was minimized by a comprehensive and systematic literature search conducted independently by 4 independent blinded authors across relevant databases (e.g., PubMed, Scopus, Web of Science) using predefined inclusion and exclusion criteria to ensure that all relevant studies were considered. In addition, prior to the search, clear inclusion and exclusion criteria were established to avoid selective reporting and ensure that only studies meeting the expected quality standards were included. Once collected, each study was critically reviewed independently by 3 authors prior to data extraction. Data extraction was standardized to ensure consistency and minimize subjectivity, and all pre-selected metabolic outcomes in the included studies were reported to prevent selective reporting. In addition, each study was evaluated based on key criteria, including study design, sample size, participant age, duration, dietary intervention details, and metabolic outcome measures, ensuring consistency in evaluating study reliability, minimizing subjectivity in data interpretation, and enhancing the overall robustness of the reported findings.

Studies were also assessed for the type of statistical analysis employed: intention-to-treat (ITT), per-protocol (PP), or as-treated (AT) (detailed in Table 2). ITT includes all randomized participants regardless of adherence, PP includes only those who completed the intervention as originally assigned, and AT analyzes outcomes based on the intervention actually received. Identifying the analytical approach is essential, as it influences the internal validity of the study and the extent to which the findings can be extrapolated to broader clinical contexts.

### 2.6. Data Synthesis and Analysis

The reporting in this review was based on the Preferred Reporting Items for Systematic Reviews and Meta-Analyses (PRISMA) guidelines. The gathered data are summarized in a series of tables and compared with respect to the category of metabolic outcomes and the effect of intervention with a defined model of dietary pattern while comparing pre- and post-intervention values of the primary outcomes, including measures of (1) anthropometry (2) glucose, lipid metabolism, and inflammation, and (3) liver function, are presented. In addition, a brief description was made of any reported changes in the secondary measures involving determinants of physical activity, smoking status, alcohol intake, sleep and diet quality, stress levels, mental health status, and self-reported quality of life.

The comparison between the applied models of dietary pattern on on the variables of the primary outcomes in this study is presented graphically using color coding. A *p*-value of less than 0.05 was considered significant when assessing the impact (the change) of the reported values of metabolic outcomes in the reviewed studies. Findings were interpreted and contrasted in the context of existing literature, discussing limitations and providing implications for practice and future research.

## 3. Results

**Figure 1:**
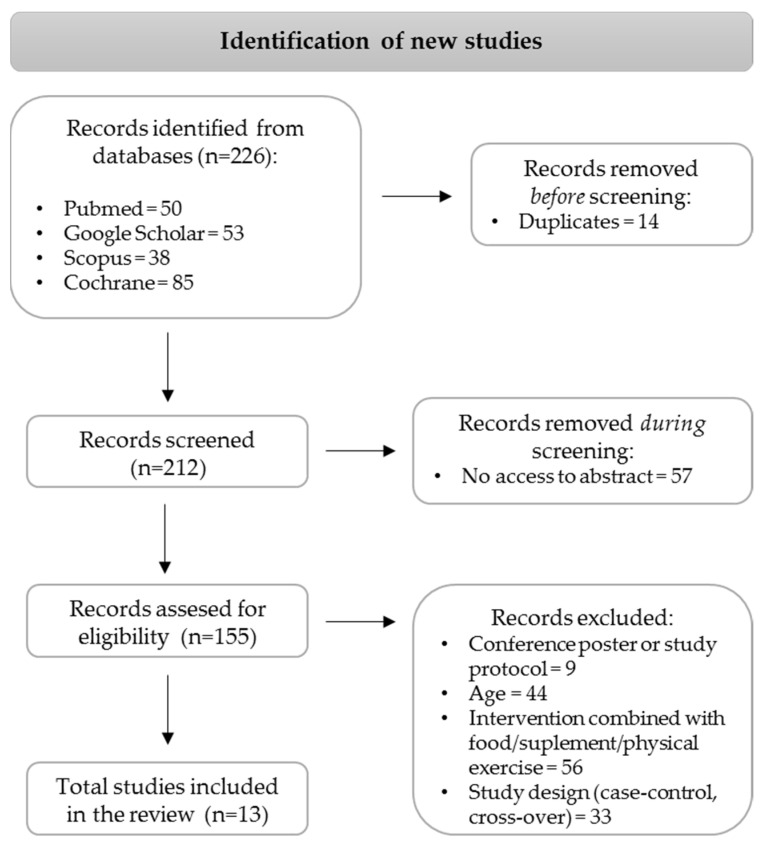
Flowchart with an overview of article identification.

The conducted electronic search initially identified 226 articles of interest; however, when considering accessibility and inclusion/exclusion criteria, 13 studies were selected and investigated in this systematic review. The flowchart of the study selection process is represented in Figure 1.

### 3.1. Study Characteristics

The characteristics of the studies and diets included in this review are detailed in Table 1 and Table 2, respectively.

Based on the characteristics of dietary pattern models used in the reviewed studies presented in Table 1, the majority of interventions used diets based on the MED pattern as the diet model [13,21,22,23] and its low-fat (LFDM) or carbohydrate (LCMD) modifications [24] (five studies), as well as the DASH diet [11,15,18,19] (three studies). On the other hand, LFD [21,22,23] (three studies) and Low-Calorie Diet (LCD) [18,19,20] (three studies) were the second choices. Furthermore, there was a single study that assessed other dietary models, such as the calorie-restricted (CR) diet [14], KD [11] and Lacto-ovo-vegetarian diet (LOV-D) [17]. The IF-based strategies included alternate-day fasting (ADF) [20] (one study) and time-restricted feeding (TRF), including TRF with protocol 16/8 [16,20] (two studies). The interventions described above were compared with the standard dietary patterns based on the official recommendations, such as American Heart Association (AHA) guidelines [13], healthy eating and weight control advice [14], and the standard weight-loss diet (SWL-D) [17]. The use of various dietary patterns resulted in differences in the macronutrient and energy intake in MASLD patients (Table 1 and Table 2). Depending on the model of the applied dietary pattern, the calorie restriction ranged from 350 to 1000 kcal/day. In terms of macronutrients, carbohydrate intake ranged from 5% in the KD to 60% in certain LFD and DASH diets; protein varied from 10 to 25% in KD and certain versions of MED, tending toward the higher end, whereas fat intake varied widely, ranging from 20 to 25% in LFD to 70% in the KD. In addition, several diets incorporated additional recommendations, including meal frequency, or encouraged higher intake of certain food groups, such as whole grains, fruits, vegetables, and lean protein (Table 1).

The comparison between studies presented in Table 2 shows that all the reviewed studies were RCTs published in the last 5 years (2016–2024). They were conducted predominantly in Iran [14,15,16,17,18,19] (six studies), with some being conducted in Australia [22,23] (two studies) and other countries, including China [20] (one study), Serbia [21] (one study), Turkey [24] (one study), Spain [13] (one study), and Thailand [11] (one study). Based on the inclusion criteria for this systematic review, all studies were conducted on adult patients diagnosed with MASLD, as well as with obesity [11,13,14,15,17,18,19,20,21] (nine studies), with some including patients with MASLD [16,23] (two studies), MASLD with T2DM/insulin resistance [22] (one study), and MASLD with insulin resistance and obesity [24] (one study).

The total sample size in the reviewed articles ranged from 22 [11] to 264 participants [20], and the mean age ranged from 23.27 ± 2.46 [13] to 53.00 ± 9.06 [23]. Most of the reviewed studies were 3 months long [14,16,17,21,22,23] (six studies); however, the duration varied between the studies and ranged from 2 [11,15,18,19,24] (five studies) to 24 months [13,20] (two studies).

The total sample assessed in this systematic review consisted of 926 participants.

### 3.2. Influence of Dietary Interventions with Model Diet Patterns on Metabolic Outcomes in MASLD Patients

#### 3.2.1. Anthropometric Outcomes

The comparison of the effects of the dietary interventions on anthropometric outcomes in MASLD patients, including body weight (BW), BMI, WC, body fat (BF), diastolic blood pressure (DBP), and systolic blood pressure (SBP), is presented in Table 3 and Table 4.

Use of the AHA diet resulted in significant reductions in BW (−4.70 ± 6.85 kg, *p* < 0.001) and BMI (−1.53 ± 2.31 kg/m^2^, *p* < 0.001); however, there were limited effects on WC and BF measures [13]. Results obtained from interventions with MED on anthropometric outcomes in MASLD differ between studies, with some indicating significant reductions in BW, BMI, WC (*p* < 0.001 for all) [21,23,24], and BF (*p* = 0.000) [21,24], as well as when LCMD and LFMD versions [24] were used. Additionally, the use of a modified form of MED, known as the FLiO diet, led to significant reductions in BW, BMI, and WC (*p* < 0.001) [13]. Prescription of TRF and ADF resulted in significant reductions in BW [16,20], with additional beneficial effects on BMI and BF reported for TRF (*p* < 0.001 for all) [16]. Prescription of the DASH diet led to significant reductions in BMI and WC (*p* < 0.001 for all) [19], along with measures of blood pressure, such as SBP and DBP (*p* = 0.002 for all) [19]. LOV-D also contributed to significant reductions in BW, BMI, and WC (*p* < 0.001 for all), along with a decrease in SBP (*p* = 0.001) [17]. There was no information available on the CR effect on evaluated anthropometric outcomes in this review (Table 3).

The comparison between the interventions demonstrated the superiority of certain dietary models over others in improving anthropometric measures in MASLD (Table 4). For example, the use of the modified MED-based intervention with the FLiO diet showed a significantly greater reduction in WC compared to the AHA (*p* = 0.021) [13], thus with no different effects on BW, BMI, and BF [13]. Nevertheless, using the MED model when compared to LFD varied in effectiveness and was reported to lead to a more pronounced effect on the WC reduction than LFD (*p* = 0.041) [23]. In contrast, the majority of MED-based interventions showed no superiority for any anthropometric outcomes [21,22] even though modified versions, LCMD and LFMD [24], were used. The use of IF-based strategies, TRF, and/or ADF, when compared to CN, has shown a superior effect on reducing BW (*p* < 0.05 for all) [16,20], as well as on BMI (*p* = 0.013) [16] and WC (*p* = 0.041) [16]. Comparison of the effects with DASH indicated that this model may have led to significantly greater reductions in BW (*p* = 0.021), BMI (*p* = 0.025), and WC (*p* = 0.002) compared to LCD [19], and also when compared to CN [15]. A superior effect of DASH in reducing BW, WC, and BF was additionally observed in contrast to the KD [11]. The LOV-D resulted in significantly greater reductions in BW, BMI, and WC (*p* < 0.001 for all) [17], along with a decrease in SBP (*p* = 0.023) when compared to SWL-D [17] (Table 4).

#### 3.2.2. Glucose and Lipid Metabolism and Inflammatory Outcomes

The comparison of the effects of the dietary interventions in MASLD patients on glucose and lipid metabolism and inflammatory outcomes, including fasting glucose and insulin, Homeostatic Model Assessment of Insulin Resistance (HOMA-IR), HbA1c, triglycerides, total cholesterol, LDL-C and HDL-C, as well as high-sensitivity C-reactive protein (hs-CRP), interleukin-6 (IL-6), monocyte chemoattractant protein-1 (MCP-1), and lipopolysaccharide (LPS), is presented in Table 5 and Table 6.

The obtained results have shown that several dietary interventions can influence metabolism and inflammatory outcomes. The use of the AHA diet led to significant improvements in glucose outcomes, including significant reductions in fasting glucose, insulin levels, and HOMA-IR (*p* < 0.005) [13]; however, it had no significant effects on lipid or inflammatory status [13]. Interventions with MED-based dietary models, such as the FLiO diet [13] and MED [21] showed certain improvements in glucose metabolism, including significant reductions in fasting glucose, insulin and HOMA-IR, as well as some favorable effects on lipid profile, including reductions in triglycerides, total cholesterol, and LDL-C [21,23], and increased HDL-C [21]. The MED also showed potential to effectively reduce the inflammatory marker hs-CRP [21]. Interestingly, the favorable effects on glucose metabolism were observed after the use of TMD, LCMD, and LFMD, which significantly reduced fasting glucose and HOMA-IR (*p* = 0.010) in MASLD patients [24]. Interventions based on IF strategies, like TRF and ADF, demonstrated mixed effects in optimizing both glucose and lipid measures [16,20]. Prescription of the DASH diet demonstrated limited impacts on fasting glucose and lipid metabolic status, although in one case, it significantly improved insulin and HOMA-IR (*p* < 0.001, for all) [15]. In addition, this dietary model was effective in reducing some inflammatory markers, including MCP-1 [19] and LPS [19]. The use of LOV-D led to significant reductions in fasting glucose, insulin, HOMA-IR, triglycerides, total cholesterol, and LDL-C [17] (Table 5).

The series of comparisons between reviewed dietary model patterns indicated distinct effects on metabolic and inflammatory markers assessed in MASLD patients (Table 6). When MED-based interventions were contrasted with LFD, the MED was significantly better in triglycerides [21] and increasing HDL-C [21], in contrast to fasting glucose and lipid status [21,22,23,24]. IF-based models, especially TRF 16/8, when compared to CN, had significantly better performance in reducing fasting glucose, LDL-C, and the inflammatory marker hs-CRP [16], whereas TRF and ADF were effective in decreasing triglyceride and total cholesterol [16,20]. The DASH diet, when compared to LCD, demonstrated superior effects on HbA1C [19], triglycerides [18], total cholesterol [18], and LDL-C [18], as well as inflammatory markers, including MCP-1 and LPS [19]. The LOV-D, when compared to SWL-D, had superior effects on reducing fasting glucose, insulin, and HOMA-IR, as well as lipid status, including a decrease in triglycerides, total cholesterol, and LDL-C levels [17]. Finally, the comparison of CR with CN was superior only in reducing total cholesterol levels [14].

#### 3.2.3. Liver Function Outcomes

The comparison of the effects of the dietary interventions in MASLD patients on liver function outcomes is presented in Table 7 and Table 8. Briefly, several dietary interventions were able to significantly influence the liver enzyme levels; however, these effects varied between these interventions. Interventions with MED-based dietary models demonstrated beneficial effects on hepatic health, including significant reduction in ALT [21,22,23], IHL [23], and CAP [23]. The use of the TMD, as well as modified versions, LCMD and LFMD, resulted in significant reductions in hepatic enzymes, including ALT and AST, as well as IHL and FIB-4 (*p* = 0.010) [24]. Similar effects were observed with another MED-based dietary model, a FLiO diet, which significantly reduced ALT and FLI (*p* < 0.001) [13]. Despite limited data, the use of IF-based interventions, especially TRF, demonstrated potential to reduce AST, ALT, and IHL scores [16]. The CR intervention showed modest reductions in ALT levels and some potential to improve pathological outcomes [14] (Table 7).

Comparisons between interventions with model dietary patterns have revealed limited evidence on their effects on the Liver Function Outcomes in MASLD. Interventions based on MED demonstrated inconsistent effects on hepatic enzymes, including AST and ALT levels, when compared to LFD [21,22]. The comparison between the TMD, LCMD, and LFMD demonstrated significantly different effects, particularly on the liver enzymes, including AST, ALT, and GGT [24], as well as on the measures of FLI and FIB-4 [24]. In addition, the use of the DASH diet was superior in reducing AST measures relative to LCD [19] and KD [11].

IF approaches, a 16/8 TRF regimen, when compared to CN, led to more significant improvements in hepatic enzymes, including AST (*p* = 0.010), ALT (*p* = 0.013), and GTT (*p* = 0.026) [16], as well as in the CAP (*p* = 0.009) [16] and LSM (*p* ˂ 0.001) [16] parameters. CR demonstrated a superior effect on reducing hepatic enzymes, particularly AST and ALT, when compared to ND [14] (Table 8).

## 4. Discussion

MASLD is a complex condition with many factors implicated in its risk/development, including nutrition and immune-metabolic health status [10]. Given that existing studies demonstrated a link between MASLD and unhealthy dietary habits [25], showing that consumption of ultra-processed foods [25,26], which is also associated with obesity and T2DM, is a considerable risk factor for developing MASLD [26] and its severity [26]. Consequently, investigating the influence and effectiveness of dietary patterns in improving hepatic function, along with immuno- metabolism, can be important in the MASLD management. Based on the identified review of 13 RCTs with dietary models and focusing on their impact on anthropometric, immune-metabolic and hepatic outcomes in MASLD patients with co-existing conditions, including obesity, MS, and T2DM, this systematic review demonstrated that interventions with model dietary patterns, especially those lasting between 2 to 3 months and based on MED and/or using TRF, have promising potential to significantly change/improve anthropometric, metabolic, and hepatic biomarkers.

MED is a model of dietary pattern originating from the traditional eating habits of countries located within the Mediterranean Sea area, recognized for its low incidence and mortality from cardiovascular conditions [27]. The MED diet is characterized by a high intake of plant-derived foods, mostly vegetables, fruits, whole grains, legumes, nuts, and seeds, along with olive oil, and moderate intakes of dairy, fish and red wine; while reducing added sugars, red meat, and refined carbohydrates [28]. The unique composition of MED, being rich in nutritional dense foods but relatively low in calories [29], makes it considered a healthy eating pattern suitable for chronic disease prevention, with implications for MASLD management [30]. There is accumulating evidence showing that a higher adherence to MED can be associated with a lower risk of developing MASLD among overweight/obese adults [31] and a lower prevalence of hepatic steatosis in adults [32], being largely explained by adiposity. In addition, a higher MedDietScore was correlated with lower insulin resistance among patients with MASLD [33], and lower measures of BW, BMI, WC [34], and BP [35]. These beneficial effects are consistent with the findings of this systematic review, indicating that interventions based on MED, as well as its modified versions, including LCMD, LFMD, and FLiO [13,21,23,24], may be effective in the reduction of anthropometric outcomes (e.g., BW, BMI, and WC), as well as positively influence glycemic metabolism and inflammatory status, leading to significant improvements in fasting glucose, insulin, HOMA-IR, and hs-CRP levels. These anti-obesogenic and anti-inflammatory properties of MED resultant from its unique composition promoting the consumption of foods rich in dietary fiber and polyphenols [36], such as fruits, vegetables, and olive oil, which all together may promote sustainable weight loss and reduce central adiposity [37,38]. Despite the limited data on liver function outcomes, the results of this review provided early indication of the promising potential of MED-based interventions, including LCMD and LFMD, to improve hepatic outcomes by decreasing liver enzymes (e.g., AST, ALT), as well as certain biomarkers associated with MASLD severity (e.g., FIB-4, LSM, and IHL). These observations are consistent with both epidemiological and clinical evidence, as the use of MED-based models have been associated with significant improvements in hepatic function, demonstrated by a reduction in IHL% [36] and decreased liver stiffness [36]. Interestingly, adherence to a higher MedDietScore was inversely associated with hepatic enzyme (ALT) and histological characteristics of severe steatosis [31]. Interestingly, enriching the MED diet with polyphenols from nuts and green plants (28 g/day of walnuts, 3–4 cups per day of green tea, 100 g per day of Mankai strain, and a green shake) enhanced the anti-inflammatory and antioxidant properties of green-MED, which were superior in reducing IHF% when compared to TMED [36]. Although these findings may suggest the potential of MED-based interventions in MASLD management [36], the MED protocols require further optimization, as some studies comparing the effectiveness of MED vs. LFD in MASLD [21,22] failed to demonstrate differences in anthropometric, lipid, and liver outcomes [22].

The results of this review demonstrated that the use of IF-based approaches, such as ADF and TRF, including the 16/8 protocol [16,20], might be effective in improving anthropometric and immune-metabolic outcomes, characterized by a reduction in BW, BMI, BF, and WC, along with optimization of fasting glucose, triglycerides, LDL-C, and hs-CRP when compared to CR [16]. Furthermore, 16/8 TRF significantly contributed to the improvements in liver function, demonstrated a reduction in hepatic enzymes (e.g., AST and ALT) [16] along with reduced liver stiffness (e.g., CAP, LSM) [16]. Nevertheless, in this analysis, the use of TRF, rather than ADF, had more pronounced effects on the cardiometabolic outcomes in MASLD patients [20]. This finding is consistent with previous studies showing that 16/8 TRF, by limiting the time-of-eating window, might be effective in controlling caloric intake among overweight/obese individuals [39] without compromising the diet quality, as consumption of all food groups was allowed [40]. Furthermore, studies evaluating IF-based strategies have linked them with certain metabolic effects, including improvement in insulin resistance and inhibition of hepatic lipogenesis [10], along with other reported improvements in the glycemic response [41] and blood pressure [42]. These beneficial effects were attributed to the regulatory role of TRE on the enzymatic activity involved in glucose metabolism, which contributes to an increase in glucose utilization, thereby leading to a metabolic switch from glucose to ketones during fasting periods, finally resulting in decreased fat accumulation in hepatic tissues. In addition, TRE may also affect the genetic expression of genes involved in both glucose and lipid homeostasis leading to reduced lipid storage and increased triglyceride utilization, which all together suggests the potential effectiveness of TRE in managing MASLD [43]. Although these studies may indicate the usefulness of TRE in MASLD management, there were studies presenting inconsistent effects of TRE on metabolism, especially when assessing the impact of this IF-based strategy on immune-metabolic status (e.g., insulin sensitivity, lipid profile, and inflammatory markers) [44,45,46], as some of them demonstrated no impact of TRF on fasting glucose, insulin, and lipid status [47,48]. Consequently, the use of IF-based strategies as a form of calorie restriction in improving MASLD outcomes requires further attention to enhance effectiveness while ensuring the safety of their protocols in this patient group.

Finally, the use of other model dietary patterns, such as DASH and LOV-D, demonstrated potential to significantly reduce anthropometric measures (e.g., BW, BMI, WC), with additional benefits in decreasing blood pressure and improving immuno-metabolic parameters, such as fasting glucose, insulin levels, HOMA-IR, triglycerides, and hs-CRP, especially for those who received DASH [15,19]. Similar effects on reduced fasting glucose, insulin levels, and triglycerides, with further improvements in the cholesterol profile, were reported for LOV-D [17]. In this study, both DASH [15,19] and LOV-D [17] were reported to positively influence liver function by reducing hepatic enzymes (e.g., ALT and AST), along with IHL% and fibrosis biomarkers (e.g., FIB-4). In previous research, interventions with DASH, by promoting intake of foods rich in protein, fiber, calcium, magnesium, potassium, zinc, and folate, while limiting those high in sodium (<2400 mg/day) and saturated fat, can contribute to the reduction of obesity and hypertension [30] and have been associated with a lower incidence of T2DM [49] and MASLD risk [50]. In this review, interventions with DASH have been shown to improve management of BP, BW, insulin homeostasis, and lipid profiles [30], as both the short/long-term prescription of low-calorie DASH were reported to be more effective in the reduction of BW, BMI, and WC when compared to standard weight loss advice given to overweight/obese adults [30], whereas intervention with DASH can contribute to the reduction of SBP and DBP, especially in those with high blood pressure or BMI at baseline [51]. Interestingly, an 8-week-long RCT conducted in overweight/obese patients diagnosed with MASLD has been shown to lead to significant beneficial effects on BW, hepatic enzymes, and immune-metabolic outcomes, including insulin sensitivity, lipid profiles, and inflammatory markers, when compared to LCD [15].

This systematic review has several advantages, including rigorous methodology, comprehensive scope, and recent data with potential clinical relevance. By employing a standardized methodology for systematic research, this work employs a structured and transparent approach to data collection, which enhances the reliability and reproducibility of findings. In addition, this study collects recent RCTs with a diverse range of interventions based on model dietary patterns exclusively in patients with MASLD and common comorbidities, which may offer evidence-based insights that can guide dietary recommendations for individuals at risk of liver and metabolic conditions. Nevertheless, the explorative approach of this review has several limitations, including a relatively low number of included RCTs and consequently a small total sample of patients with MASLD with measured immuno-metabolic and hepatic outcomes. Despite the systematic approach and predetermined inclusion criteria being focused only on adults with MASLD and comorbidities, like obesity, MS, and T2DM, the significant heterogeneity in sample size, duration, and type of intervention, as well as individual characteristics, including nationality/geographical backgrounds and age, may complicate the comparison of the effectiveness of the interventions and the generalizability of the findings to broader populations. Although this review strived to include high-quality RCTs, these variations in study design, such as the absence of robust blinding procedures or control groups, along with lack of or duration of follow-up periods, restrict the ability to assess the long-term efficacy and sustainability of dietary approaches in MASLD management. While efforts were made to control for baseline metabolic differences, unmeasured confounders, such as lifestyle habits, medication use, and genetic predispositions, may additionally influence the results. Another limitation is the limited data on hepatic outcomes, which were not always available for all reviewed RCTs, and the improvement in liver function parameters was, in the majority, based on non-invasive biomarkers, and there is no histological confirmation. These issues may introduce a risk of bias and limit the ability to determine the effectiveness of dietary intervention on liver function. In addition, the papers were evaluated by non-English speakers, as the authors are proficient in the respective language (e.g., Polish or Spanish), which may pose a risk of selection or interpretation bias, as the evaluation was not cross-checked by multiple reviewers with proficiency in the same language. Despite this drawback, the authors made relevant efforts to ensure a rigorous assessment by adhering to standardized criteria. 

Given the limitations of the current evidence, further research is essential to confirm and expand upon the preliminary findings presented in this systematic review. Future large-scale, long-term RCTs are necessary to evaluate the sustained efficacy and safety of dietary interventions, particularly MED and IF approaches. These studies should be designed with rigorous methodologies and standardized dietary protocols to minimize bias and improve comparability across trials. Additionally, research focused on the mechanisms behind the beneficial effects of these interventions—such as their impact on hepatic steatosis, inflammation, insulin resistance, and gut microbiota composition—will provide deeper insights into the role of nutrition in managing MASLD.

In light of the clinical relevance of the findings presented here, medical practitioners may consider incorporating MED- and IF-based dietary strategies into the management of MASLD, especially in patients with comorbid conditions such as obesity, T2DM, and MS. These approaches have demonstrated significant improvements in BW, BMI, WC, and key metabolic markers, particularly in interventions lasting more than three months. The promising effectiveness of these strategies emphasizes the need for personalized dietary recommendations tailored to individual patient profiles. This approach, combined with the implementation of standardized treatment protocols, holds potential for improving clinical outcomes in MASLD management.

## 5. Conclusions

The main findings demonstrated the potential effectiveness of MED-based and IF-based strategies, such as TRF, with the most pronounced effects observed in studies lasting 2–3 months. Several dietary models, including MED, IF, DASH, and LOV-D, demonstrated substantial reductions in BW, BMI, and WC, whereas others, being predominantly a modified MED-based and TRF approaches, additionally contributed to significant improvements in glycemic, lipid, and inflammatory status, as well as hepatic function. However, given the limited number of studies, with some being inconsistent with reported results, more investigations are needed to evaluate the effects of long-term adherence and sustainability of different dietary interventions for MASLD, particularly comparing the MED, DASH, and IF-based approaches. Additionally, these trials should explore the optimal macronutrient composition, individual variability in dietary response, and the underlying mechanisms linking these diets to hepatic and metabolic improvements.

## Figures and Tables

**Table 1:** Description of dietary interventions and applied diet models used in MASLD patients.

Name of Dietary Pattern Model	Macronutrient Distribution (% of Total Kcal Consumed a Day)			Additional Recommendations
	Carbohydrates	Protein	Fat	
American Heart Association (AHA) guidelines [13]	55	15	30	Prescribed 3–5 meals per day.
Healthy eating and weight control advice (normal diet, ND) [14] 11 March 2025 13:45:00	53	17	30	The diet was based on the Food Guide Pyramid, along with the recommendations of the Obesity Education Initiative Expert Panel.
Control Normal (CN) diet [15,16]	52–55	16–18	30	The diet was based on the foods, including daily intake of (servings) grains (10), simple sugars (5), vegetables (4) and fruits (4), dairy (2), meats (including 2 servings of lean meat), poultry and fish (4), nuts, seeds and legumes (1) and fats/oils (3).
	55	15	30	Normal usual diet required adherence to the caloric, macronutrient, and fruit and vegetable consumption, including 9 servings of fruits and vegetables.
Standard weight-loss diet (SWL-D) [17]	50–55	15–20	25–30	Follow the standard food pyramid, which was free of all sources of food
				Protein sources (18%) are provided by meat and meat products, poultry, fish and seafood, and the flesh of any other animals.
Calorie-restricted (CR) diet [14]	53	17	30	The diet was planned individually based on the optimal servings of each food group from the Food Guide Pyramid, while accounting for food preferences and allergies, or intolerances.
				Diet restricted calorics intake from 500 to 1000 kcal/day, deducted from the estimated energy requirements.
Low-calorie diet (LCD) [18,19,20]	55–60	10–15	30	The diet was based on the food-based dietary guidelines for Iranians and included a variety of raw and cooked vegetables, fruits, and legumes consumed daily, along with regular intake of dairy products, such as milk, cheese, and yogurt, and emphasis on whole-grain breads and cereals. Animal protein sources include lean meats, chicken, fish, and eggs. Fats and oils are used in moderation, while the consumption of sugar, salt, and processed foods is limited. Water and unsweetened beverages are prioritized.
				Diet-restricted calorics intake of 500 kcal/day deducted from the estimated energy requirements.
	N/A	N/A	N/A	A diet with reduced caloric intake to 80% of daily requirements, and free from any restrictions in food group intake.
Low-Fat Diet (LFD) [21,22,23]	60	15	25	A diet with reduced calorie intake providing 600–800 kcal less than the daily energy requirement.
				A balanced diet that focused on the intake of low-glycemic carbohydrate sources.
				The intake of all types of fats from both animal and vegetable sources is reduced.
	50	20	30	The diet was based on recommendations of the Australian Dietary Guidelines and The Heart Foundation.
				A balanced diet promoting the intake of a variety of vegetables, fruits, whole grains, lean proteins, and dairy or dairy alternatives, limiting saturated fat, added sugars, and salt. The main fat sources are nuts, seeds, and avocados. Regular consumption of fish, legumes, and lean meats is recommended. Water intake is prioritized, while other beverages and alcoholic drinks are consumed in small portions occasionally.
	50	20	30	The diet was based on National Health and Medical Research Council and American Heart Association Dietary recommendations.
				The diet included a monthly intake of 1 kg of natural muesli and 200 g of low-fat snack bars, limiting a saturated fat to <10% of total calorie intake.
Ketogenic diet (KD) [11]	5	25	70	A low-carbohydrate, high-protein, high-fat diet providing 1500 kcal a day.
Dietary Approaches to Stop Hypertension (DASH) diet [11,15,18,19]	55–60	10–15	30	A DASH diet with reduced calorie intake providing l500 kcal less than the daily energy requirement.
				The diet was based on intake of whole grains, fruits, vegetables, dairy, and plant-based proteins, while limiting saturated fat, added sugar, and alcohol.
				The diet included at least 4–5 servings of whole grains, 3–4 servings of vegetables and fruits, and 2 servings of dairy. Saturated fat was reduced to <5% of total energy intake, added sugars to ≤3%. Alcohol consumption was restricted to 2 drinks/day for men and 1 for women.
	60	15	25	General lifestyle advice counseling DASH as low-fat, rich in vegetables/fruits and whole grains providing 1500 kcal a day, being rich in foods that are high in potassium, calcium, and magnesium.
	52–55	16–18	30	A DASH diet with reduced calorie intake provides 350–700 kcal less than the daily energy requirement.
				The diet was rich in fruits, vegetables, whole grains, and low-fat dairy products, and low in saturated fats, cholesterol, refined grains, and sweets.
				The diet included a daily intake (servings) of grains (8) with at least 3 servings from whole grains, simple sugars (2), vegetables (5) and fruits (6), low-fat dairy (3), meats (including 4 servings of lean meat), poultry and fish (4), nuts, seeds and legumes (2) and fats/oils (3). Sodium consumption was limited to <2400 mg/day.
Lacto-ovo-vegetarian diet (LOV-D) [17]	50–55	15–20	25–30	The diet eliminated intake of meat, poultry, and fish, while promoting consumption of dairy products and eggs as the main protein sources.
				The key protein sources were eggs (24%), dairy (19%), soy (16%), nuts (8%), vegetables, and fruits (7%).
Alternate-day fasting (ADF) [16,20]	55	15	30	The diet was scheduled to reduce calorie intake by 25% on the fast day (24 h), and allowed for ad libitum intake of all foods on the feed day (24 h). The feed and fast days began at midnight, and all fast day meals were consumed between 12.00 p.m. and 2.00 p.m.
				Fast day meals were provided as a 3-day rotating menu and were based on the American Heart Association (AHA) guidelines.
Time-Restricted Feeding (TRF) 16/8 [20]	55	15	30	The diet was scheduled to achieve 16 consecutive hours of fasting from all caloric intake and 8 h of eating time each day.
				Free sugar intake was reduced to 3% of daily calorie intake.
	55	15	30	The diet provided meals within an 8-h window, which was followed by refraining from consumption of all food or beverages that included energy for 16 h.
				The amount or type of food consumed during the 8-h window, as well as the timing of the feeding window during the day, were freely chosen to accommodate lifestyle habits.
Mediterranean (MED) diet [13,21,22,23]	40–45	25	30–35	The Fatty Liver in Obesity (FLiO) Diet is a Mediterranean-based diet adapted for Fatty Liver in Obesity study.
				Diet based on high intake of extra virgin olive oil and omega 3 fatty acids, as well as low in glycemic index carbohydrates; while reducing saturated fats, trans fats, and cholesterol.
	33	15–20	44	The diet was based on the traditional Cretan diet and included a high intake of vegetables, including leafy greens, tomatoes, onions, and garlic; legumes (2 servings/week), along with a moderate intake of dairy, preferably fermented varieties like yogurt. The diet included fish or shellfish (3 servings/week), especially oily fish as a source of omega-3 fatty acids, whereas consumption of red meat was limited to small portions, with a preference for lean, grass-fed options. Sweets and sugary drinks are avoided, and red wine is consumed in moderation.
				The diet was considered as high-fat, with extra virgin olive oil as the main fat (50% from monounsaturated fatty acids), along with antioxidants.
	50	15	25	A MED diet with reduced calorie intake providing 600–800 kcal less than the energy requirement.
				The diet is based on vegetables, along with the intake of poultry and fish, while reducing red meat consumption. Animal fats from butter, cream, and lard were not allowed.
	40	20	35–40	The diet was based on traditional Cretan diet with added adjustments to the amount of protein intake (standardization of protein with LCD).
				The saturated fat was reduced to <10% of total kcal consumed daily.
				The diet included intake of 750 g of nuts (almonds or walnuts) and 750 mL of olive oil monthly.
Low carbohydrate Mediterranean diet (LCMD) [24]	≤35	15–20	>45	All MD diet types had a reduced intake of saturated fat to <10%, and included consumption of at least 3 portions of vegetables and 2–4 portions of fruits a day, and fish twice a week. The meal pattern was determined to provide 3 main meals and 2–3 snacks a day.
Low-fat Mediterranean diet (LFMD) [24]	≥55	15–20	20–25	
Typical Mediterranean diet (TMD) [24]	40–45	15–20	35–40	

N/A, Not Applicable.

**Table 2:** Main characteristics of the RCT studies included in the systematic review.

Reference	Country	Population	Final Participants Numbers	Age (Mean ± SD)	Dietary Intervention		Type of Analysis
					Type	Duration (Months)	ITT; PP or AT
Kestane and Bas (2024) [24]	Turkey	Patients with MASLD with obesity and insulin resistance	LFMD: 21	LFMD: 39.48 ± 9.17	Low-fat Mediterranean diet (LFMD)	2	PP
			LCMD: 21	LCMD: 39.71 ± 10.34	Low-carbohydrate Mediterranean diet (LCMD)		
			TMD: 21	TMD: 39.48 ± 9.17	Typical Mediterranean diet (TMD)		
Mogna-Pelaez et al. (2024) [13]	Spain	Patients with MASLD and obesity	AHA: 48 (follow up: 32)	AHA: 33.7 ± 4	American Heart Association (AHA) diet	24	PP
			FLiO: 50 (follow up: 26)	FLiO: 33.3 ± 4	Mediterranean-based diet deisgned for the Fatty Liver in Obesity study (FLiO) diet		
			C: 45	C: 23.27 ± 2.46	No Diet		
Kord-Varkaneh et al. (2023) [16]	Iran	Patients with MASLD	TRF: 22	TRF: 41.36 ± 10.5	Time-restricted feeding (16/8) (TRF) with a low-sugar diet	3	PP
			CN: 23	CN: 44.17 ± 4.9	Control Normal (CN) diet		
Garousi et al. (2023) [17]	Iran	Patients with MASLD and obesity	LOV-D: 37	LOV-D: 43.51 ± 9.85	Lacto-ovo-vegetarian diet (LOV-D)	3	PP
			SWL-D: 38	SWL-D: 42.84 ± 9.85	Standard weight-loss diet (SWL-D)		
Rooholahzadegan et al. (2023) [19]	Iran	Patients with MASLD and obesity	DASH: 20	DASH: 38.80 ± 9.98	Dietary Approaches to Stop Hypertension (DASH) diet	2	PP
Badali et al. (2023) [18]			LCD: 20	LCD: 37.10 ± 9.74	Low-calorie diet (LCD)		
Sakkarin Chirapongsathorn et al. (2023) [11]	Thailand	Patients with MASLD and obesity	DASH: 10	DASH: 40.2 ± 7.57	Dietary Approaches to Stop Hypertension (DASH) diet	2	PP
			KD: 12	KD: 37.42 ± 7.49	Ketogenic diet (KD)		
Asghari et al. (2022) [14]	Iran	Patients with MASLD and obesity	CR: 30	CR: 40.08 ± 7.08	Calorie-restricted (CR) diet	3	PP
			ND: 30	ND: 39.27 ± 5.51	Healthy eating and weight control advice (normal diet, ND)		
George et al. (2022) [22]	Australia	Patients with MASLD and T2DM/insulin resistance	MED: 18	MED: 52.6 ± 11.7	Mediterranean (MED) diet	3	ITT
			LCD: 21	LCD: 52.1 ± 13.6	Low-calorie diet (LCD)		
Ristic-Medic et al. (2020) [21]	Serbia	Patients with MASLD and obesity	MED: 12	MED: 34.42 ± 4.66	Calorie-restriced Mediterranean diet (MedDiet)	3	PP
			LFD: 12	LFD: 32.92 ± 3.78	Calorie-restriced low-fat diet (LFD)		
Cai et al. (2019) [20]	China	Patients with MASLD and obesity	ADF: 95	ADF: 35.50 ± 4.417	Alternate-day fasting (ADF)	24	PP
			TRF: 90	TRF: 33.56 ± 6.23	Time-Restricted Feeding (TRF) 16/8		
			LCD: 79	LCD: 34.54 ± 6.96	Low-calorie diet (LCD)		
Properzi et al. (2018) [23]	Australia	Patients with MASLD	MED: 24	MED: 51.00 ± 13.36	Mediterranean (MED) diet	3	ITT
			LCD: 24	LCD: 53.00 ± 9.06	Low-calorie diet (LCD)		
Razavi Zade et al. (2016) [15]	Iran	Patients with MASLD and obesity	DASH: 30	DASH: 39.7 ± 7.3	Dietary Approaches to Stop Hypertension (DASH) diet	2	ITT
			CN: 30	CN: 42.8 ± 10.6	Control Normal (CN) diet		

ITT, intention to treat; PP, per protocol; AT, as treated.

**Table 3:** Changes in anthropometric outcomes upon intervention with dietary pattern models in RCT studies conducted in MASLD patients.

Dietary Pattern Model		Anthropometric Outcomes																	
		BW			BMI			WC			BF			DBP			SBP		
		B	PI	*p*	B	PI	*p*	B	PI	*p*	B	PI	*p*	B	PI	*p*	B	PI	*p*
AHA	Mogna-Pelaez et al. (2024) [13]	65.61 ± 12.01	−4.70 ± 6.85 ^	*p* < 0.001	23.27 ± 2.46	−1.53 ± 2.31 ^	*p* < 0.001	78.78 ± 9.10	−2.83 ± 8.28 ^	NS	19.30 ± 5.70	−1.55 ± 3.24 ^	NS	N/A	N/A	N/A	N/A	N/A	N/A
FLiO diet	Mogna-Pelaez et al. (2024) [13]	65.61 ± 12.01	−7.31 ± 5.82 ^	*p* < 0.001	23.27 ± 2.46	−2.49 ± 2.00 ^	*p* < 0.001	78.78 ± 9.10	−6.67 ± 6.35 ^	*p* < 0.001	19.30 ± 5.70	−1.85 ± 4.13 ^	NS	N/A	N/A	N/A	N/A	N/A	N/A
MED	George et al. (2022) [22]	87.7 ± 21.1	88.1 ± 21.9	*p* = 0.45	31.6 ± 5.4	31.5 ± 5.5	*p* = 0.63	105.1 ± 14.7	106.2 ± 15.2	*p* = 0.42	39.1 ± 7.9	38.9 ± 7.7	*p* = 0.96	82.8 ± 7.1	83.7 ± 8.1	*p* = 0.74	125.4 ± 12.1	123.3 ± 13.4	*p* = 0.52
	Properzi et al. (2018) [23]	89.3 ± 12.7	87.3 ± 12.5	*p* < 0.001	31.8 ± 4.0	31.1 ± 4.0	*p* < 0.001	105.6 ± 10.3	102.9 ± 10.4	*p* = 0.001	N/A	N/A	N/A	81 ± 7	78 ± 8	*p* = 0.07	126 ± 14	122 ± 13	*p* = 0.09
	Ristic-Medic et al. (2020) [21]	101.11 ± 9.09	91.88 ± 9.48	*p* = 0.000	30.43 ± 1.81	27.65 ± 1.80	*p* = 0.000	105.67 ± 5.94	95.83 ± 5.73	*p* = 0.000	26.17 ± 1.71	21.27 ± 3.05	*p* = 0.000	N/A	N/A	N/A	N/A	N/A	N/A
TMD	Kestane and Bas (2024) [24]	91.90 ± 10.94	84.43 ± 10.34	*p* = 0.00	32.30 ± 1.08	29.74 ± 1.22	*p* = 0.00	109.93 ± 7.40	100.52 ± 7.35	*p* = 0.00	40.14 ± 6.14	35.52 ± 6.58	*p* = 0.00	N/A	N/A	N/A	N/A	N/A	N/A
LCMD		93.71 ± 12.36	86.87 ± 12.15	*p* = 0.00	32.46 ± 1.68	30.08 ± 1.67	*p* = 0.00	114.71 ± 14.78	106.10 ± 13.81	*p* = 0.00	38.97 ± 6.22	35.24 ± 5.94	*p* = 0.00	N/A	N/A	N/A	N/A	N/A	N/A
LFMD		94.12 ± 11.44	86.71 ± 10.87	*p* = 0.00	32.34 ± 1.19	29.80 ± 1.23	*p* = 0.00	111.19 ± 6.55	102.21 ± 6.81	*p* = 0.00	37.99 ± 7.96	33.92 ± 8.61	*p* = 0.00	N/A	N/A	N/A	N/A	N/A	N/A
LFD	George et al. (2022) [22]	89.8 ± 24.4	86.6 ± 19.6	*p* = 0.11	32.7 ± 6.9	31.3 ± 4.9	*p* = 0.16	108.9 ± 20.6	105.1 ± 14.7	*p* = 0.70	40.8 ± 7.9	38.9 ± 7.9	*p* = 0.35	83.3 ± 9.8	80.3 ± 8.6	*p* = 0.21	127.4 ± 19.2	118.6 ± 10.8	*p* = 0.033
	Properzi et al. (2018) [23]	81.3 ± 13.3	79.6 ± 13.5	*p* = 0.001	30.1 ± 5.69	29.5 ± 5.8	*p* = 0.001	98.0 ± 12.0	93.9 ± 10.6	*p* < 0.001	N/A	N/A	N/A	79 ± 9	76 ± 9	*p* = 0.07	130 ± 16	126 ± 14	*p* = 0.08
	Ristic-Medic et al. (2020) [21]	102.12 ± 8.19	92.41 ± 8.14	*p* = 0.000	30.17 ± 2.28	27.68 ± 2.44	*p* = 0.000	107.58 ± 6.96	98.83 ± 8.04	*p* = 0.000	26.93 ± 3.50	21.86 ± 3.95	*p* = 0.000	N/A	N/A	N/A	N/A	N/A	N/A
LCD	Badali et al. (2023) [18]	93.49 ± 13.98	87.88 ± 13.88	*p* < 0.001	34.02 ± 3.61	31.96 ± 3.57	*p* < 0.001	109.92 ± 9.80	105.0 ± 9.60	*p* < 0.001	N/A	N/A	N/A	82.78 ± 11.27	77.35 ± 9.54	*p* = 0.044	125.50 ± 11.91	120.25 ± 13.52	*p* = 0.022
	Rooholahzadegan et al. (2023) [19]																		
	Cai et al. (2019) [20]	72.94 ± 8.00	71.77 ± 6.90	N/A	26.34 ± 2.73	26.64 ± 1.66	N/A	92.59 ± 4.98	88.54 ± 5.10	N/A	29.06 ± 3.64 ***	28.01 ± 3.49 ***	N/A	N/A	N/A	N/A	N/A	N/A	N/A
TRF 16/8	Cai et al. (2019) [20]	74.98 ± 8.02	71.67 ± 7.37	*p* < 0.05	26.76 ± 1.59	26.60 ± 1.46	N/A	91.54 ± 4.43	87.58 ± 4.39	N/A	30.27 ± 3.23 ***	27.65 ± 3.34 ***	N/A	N/A	N/A	N/A	N/A	N/A	N/A
	Kord-Varkaneh et al. (2023) [16]	83.75 ± 12.71	80.54 ± 12.12	*p* < 0.001	29.13 ± 2.64	28.02 ± 2.65	*p* ˂ 0.0001	104.59 ± 10.47	101.91 ± 7.42	*p* = 0.042	26.69 ± 5.35 ***	24.22 ± 4.88 ***	*p* = 0.001	N/A	N/A	N/A	N/A	N/A	N/A
ADF	Cai et al. (2019) [20]	75.32 ± 8.53	71.28 ± 7.02	N/A	26.12 ± 2.21	26.15 ± 1.58	N/A	92.07 ± 5.29	87.19 ± 4.88	N/A	30.58 ± 3.95 ***	27.10 ± 2.52 ***	N/A	N/A	N/A	N/A	N/A	N/A	N/A
LOV-D	Garousi et al. (2023) [17]	87.16 ± 18.15	81.30 ± 17.20	*p* < 0.001	32.02 ± 4.57	29.88 ± 4.54	*p* < 0.001	109.97 ± 12.29	102.43 ± 11.68	*p* < 0.001	N/A	N/A	N/A	9.82 ± 0.71	9.52 ± 0.75	*p* = 0.090	12.52 ± 1.34	11.86 ± 1.14	*p* = 0.001
SWL-D	Garousi et al. (2023) [17]	85.97 ± 15.46	83.77 ± 15.40	*p* < 0.001	30.06 ± 3.8	29.33 ± 3.98	*p* < 0.001	108.39 ± 10.91	106.01 ± 10.81	*p* < 0.001	N/A	N/A	N/A	10.03 ± 0.97	9.73 ± 0.87	*p* = 0.045	13.03 ± 1.37	12.72 ± 1.28	*p* = 0.58
DASH	Badali et al. (2023) [18]	93.32 ± 19.51	85.57 ± 18.62	*p* > 0.001	33.43 ± 4.09	30.64 ± 4.06	*p* < 0.001	111.25 ± 12.29	103.32 ± 12.67	*p* < 0.001	N/A	N/A	N/A	83.50 ± 10.89	75.25 ± 5.73	*p* = 0.002	131.25 ± 10.11	119.00 ± 9.54	*p* = 0.002
	Rooholahzadegan et al. (2023) [19]																		
	Sakkarin Chirapongsathorn et al. (2023) [11]	78.33 ± 12.66	76.1 ± 12.1	N/A	31.16 ± 3.93	29.6 ± 3.2	N/A	96.83 ± 12.95	92.6 ± 10.9	N/A	31.4 ± 8.1 ***	30.07 ± 7.2 ***	N/A	85.8 ± 10.6	83.9 ± 13.3	N/A	130.8 ± 15.8	135.2 ± 14.07	N/A
	Razavi Zade et al. (2016) [15]	81.0 ± 8.9	77.2 ± 7.9	NS	28.5 ± 3.2	27.2 ± 2.9	NS	99.3 ± 8.4	95.1 ± 7.7	NS	N/A	N/A	N/A	N/A	N/A	N/A	N/A	N/A	N/A
KD	Sakkarin Chirapongsathorn et al. (2023) [11]	82.99 ± 11.12	76.8 ± 11.4	N/A	31.93 ± 2.7	29.4 ± 2.9	N/A	102.44 ± 9.67	93.5 ± 5.5	N/A	34.2 ± 5.8 ***	29.3 ± 6.6 ***	N/A	84.1 ± 11.9	79.3 ± 11.06	N/A	132.3 ± 12.2	122.9 ± 11.2	N/A
CR	Asghari et al. (2022) [14]	89.62 ± 14.20	N/A	N/A	31.32 ± 3.31	N/A	N/A	N/A	N/A	N/A	N/A	N/A	N/A	N/A	N/A	N/A	N/A	N/A	N/A
ND	Asghari et al. (2022) [14] 3 November 2024 13:45:00	86.61 ± 10.70	N/A	N/A	30.41 ± 3.39	N/A	N/A	101.88 ± 8.18	N/A	N/A	N/A	N/A	N/A	N/A	N/A	N/A	N/A	N/A	N/A
CN	Razavi Zade et al. (2016) [15]	77.8 ± 10.1	75.5 ± 9.3	NS	28.3 ± 3.3	27.5 ± 3.0	NS	94.9 ± 12.7	92.3 ± 12.2	NS	N/A	N/A	N/A	N/A	N/A	N/A	N/A	N/A	N/A
	Kord-Varkaneh et al. (2023) [16]	89.33 ± 18.47	88.31 ± 11.01	*p* = 0.064	30.60 ± 30.09	30.26 ± 3.08	*p* = 0.063	107.09 ± 9.47	107.19 ± 8.93	*p* = 0.928	27.21 ± 7.33 ***	28.93 ± 7.91 ***	*p* = 0.041	N/A	N/A	N/A	N/A	N/A	N/A

Data are presented as mean ± SD, or median (25th–75th). B and PI are reported as exact values, otherwise marked ^ and displayed as MD. N/A states where there was no data available. BW expressed in kilograms (kg). BMI expressed in kg/m^2^. WC expressed in cm. BF expressed as % otherwise marked *** and presented in kg. SBP and DBP are expressed as mmHg. A *p*-value > 0.05 is considered NS. BW, Body Weight; BMI, Body Mass Index; WC, Waist circumference; BF, Body Fat; DBP, Diastolic Blood Pressure; SBP, Systolic Blood Pressure; MD, Mean Difference; SD, Standard Deviation; NS, Not significant; N/A, Not Available; B, Baseline; PI, Post-intervention; AHA, American Heart Association guidelines; ND, Normal diet; CN, Control Normal diet; SWL-D, Standard weight-loss diet; CR, Calorie-restricted diet; LCD, Low-calorie diet; LFD, Low-Fat Diet; KD, Ketogenic diet; DASH, Dietary Approaches to Stop Hypertension diet; LOV-D, Lacto-ovo-vegetarian diet; ADF, Alternate-day fasting; TRF, Time-Restricted Feeding 16/8; FLiO, Fatty Liver in Obesity diet; MED, Mediterranean diet; LCMD, Low carbohydrate Mediterranean diet; LFMD, Low-fat Mediterranean diet; TMD, Typical Mediterranean diet.

**Table 4:** Comparisons of changes in anthropometric outcomes upon intervention with dietary pattern models of the RCT studies conducted in MASLD patients.

Change in the Anthropometric Outcomes Between Interventions with Dietary Model Patterns in MASLD							
Compared Dietary Models	Reference	BW	BMI	WC	BF	DBP	SBP
AHA vs. FLiO	Mogna-Pelaez et al. (2024) [13]	NS	NS	*p* = 0.021	NS	N/A	N/A
MED vs. LFD	George et al. (2022) [22]	*p* = 0.77	*p* = 0.57	*p* = 0.64	*p* = 0.49	*p* = 0.85	*p* = 0.70
	Properzi et al. (2018) [23]	*p* = 0.658	*p* = 0.608	*p* = 0.041	N/A	*p* = 0.396	*p* = 0.714
	Ristic-Medic et al. (2020) [21]	*p* = 0.342	*p* = 0.342	*p* = 0.233	*p* = 0.989	N/A	N/A
TMD vs. LCMD vs. LFMD	Kestane and Bas (2024) [24]	*p* = 0.84	*p* = 0.76	*p* = 0.74	*p* = 0.50	N/A	N/A
TRF 16/8 vs. CN	Kord-Varkaneh et al. (2023) [16]	*p* = 0.029	*p* = 0.013	*p* = 0.041	*p* = 0.410	N/A	N/A
TRF vs. CN	Cai et al. (2019) [20]	*p* < 0.001	N/A	N/A	*p* < 0.001	N/A	N/A
ADF vs. CN		*p* < 0.001	N/A	N/A	*p* < 0.001	N/A	N/A
DASH vs. LCD	Rooholahzadegan et al. (2023) [19]	*p* = 0.021	*p* = 0.025	*p* = 0.002	N/A	*p* = 0.087	*p* = 0.162
	Badali et al. (2023) [18]						
DASH vs. KD	Sakkarin Chirapongsathorn et al. (2023) [11]	*p* = 0.001	*p* = 0.17	*p* = 0.019	*p* = 0.007	*p* = 0.39	*p* < 0.001
DASH vs. CN	Razavi Zade et al. (2016) [15]	*p* = 0.006	*p* = 0.01	*p* = 0.001	N/A	N/A	N/A
LOV-D vs. SWL-D	Garousi et al. (2023) [17]	*p* < 0.001	*p* < 0.001	*p* < 0.001	N/A	*p* = 0.310	*p* = 0.023
CR vs. ND	Asghari et al. (2022) [14]	N/A	N/A	N/A	N/A	N/A	N/A

Data are presented as *p*-values and color-coded along with the level of significance in the change of reported PI values between interventions with model diets. Colors in table refer to significant (green) and not significant (red) change in the reported PI values. N/A states where there was no data available (Yellow shadowing in the table). A *p*-value > 0.05 is considered NS. BW, Body Weight; BMI, Body Mass Index; WC, Waist circumference; BF, Body Fat; DBP, Diastolic Blood Pressure; SBP, Systolic Blood Pressure; NS, Not significant; N/A, Not Available; PI, Post-intervention; AHA, American Heart Association guidelines; ND, Normal diet; CN, Control Normal diet; SWL-D, Standard weight-loss diet; CR, Calorie-restricted diet; LCD, Low-calorie diet; LFD, Low-Fat Diet; KD, Ketogenic diet; DASH, Dietary Approaches to Stop Hypertension diet; LOV-D, Lacto-ovo-vegetarian diet; ADF, Alternate-day fasting; TRF, Time-Restricted Feeding 16/8; FLiO, Fatty Liver in Obesity diet; MED, Mediterranean diet; LCMD, Low carbohydrate Mediterranean diet; LFMD, Low-fat Mediterranean diet; TMD, Typical Mediterranean diet.

**Table 5:** Changes in glucose and lipid metabolism outcomes along with inflammatory status upon intervention with dietary pattern models of the RCT studies conducted in MASLD patients.

Dietary Pattern Model		Glucose and Lipid Metabolism Outcomes Along with Inflammatory Status																								
		Glucose Metabolism												Lipid Metabolism												Inflammatory Status
		Glucose			Insulin			HOMA-IR			HbA1c			Triglycerides			Cholesterol			LDL-C			HDL-C			
		B	PI	*p*	B	PI	*p*	B	PI	*p*	B	PI	*p*	B	PI	*p*	B	PI	*p*	B	PI	*p*	B	PI	*p*	
AHA	Mogna-Pelaez et al. (2024) [13]	90.73 ± 6.49	−9.19 ± 18.29 ^	*p* < 0.005	4.16 ± 1.79	−4.81 ± 7.17 ^	*p* < 0.005	0.94 ± 0.43	−1.55 ± 2.18 ^	*p* < 0.001	5.14 ± 0.38	−0.10 ± 0.43 ^	NS	83.57 ± 38.16	−0.81 ± 71.80 ^	NS	218 ± 40.20	2.53 ± 43.75 ^	NS	133.51 ± 4.81	3.12 ± 35.60 ^	NS	67.76 ± 17.65	−0.37 ± 14.98 ^	NS	N/A
FLiO diet	Mogna-Pelaez et al. (2024) [13]	90.73 ± 6.49	−8.08 ± 11.09 ^	*p* < 0.005	4.16 ± 1.79	−5.64 ± 5.46 ^	*p* < 0.001	0.94 ± 0.43	−1.55 ± 1.54 ^	*p* < 0.001	5.14 ± 0.38	−0.08 ± 0.43 ^	NS	83.57 ± 38.16	−20.08 ± 59.01	NS	218 ± 40.20	−5.15 ± 25.66 ^	NS	133.51 ± 4.81	−3.48 ± 22.57 ^	NS	67.76 ± 17.65	3.88 ± 8.73 ^	NS	N/A
MED	George et al. (2022) [22]	5.8 ± 1.6 *	5.7 ± 1.1 *	*p* = 0.410	16.4 ± 8.9	15.5 ± 8.5	*p* < 0.740	4.4 ± 3.2	3.9 ± 2.3	*p* < 0.650	N/A	N/A	N/A	1.8 ± 0.9 *	1.7 ± 0.8 *	*p* = 0.490	5.1 ± 1.6 *	5.1 ± 1.6 *	*p* = 0.072	3.2 ± 1.3 *	3.1 ± 1.3 *	*p* = 0.487	1.2 ± 0.2 *	1.2 ± 0.3 *	*p* = 0.450	hs-CRP-B: 2.6 ± 2.3 * PI: 2.2 ± 1.9 * *p* = 0.890
	Properzi et al. (2018) [23]	100.3 ± 29.7	105.8 ± 31.7	*p* = 0.920	12.2 ± 6.69	15.33 ± 8.8	*p* = 0.330	3.91 ± 1.92	3.63 ± 1.93	*p* = 0.263	6.1 ± 1.1	5.9 ± 1.0	*p* = 0.045	165.6 ± 76.2	144.2 ± 76.2	*p* = 0.008	184.8 ± 49.9	175.2 ± 49.5	*p* = 0.010	109.4 ± 44.9	105.6 ± 43.3	*p* = 0.024	41.8 ± 8.5	41.4 ± 8.5	*p* = 0.052	N/A
	Ristic-Medic et al. (2020) [21]	5.11 ± 0.51 *	4.49 ± 0.68 *	*p* = 0.000	17.70 ± 3.24 *	13.67 ± 2.94 *	*p* = 0.000	3.96 (3.40–4.76) ^	2.63 (2.28–3.04) ^	*p* = 0.000	N/A	N/A	N/A	1.92 (1.35–2.55) *^	1.06 (0.80–1.20) *^	*p* = 0.000	6.00 ± 0.78 *	4.83 0.95 *	*p* = 0.000	3.67 ± 0.72 *	2.88 ± 0.82 *	*p* = 0.000	1.29 ± 0.13 *	1.41 ± 0.15 *	*p* = 0.000	hs-CRP-B: 1.02 (0.75–2.23) ^ PI: 0.81 (0.34–1.40) ^ *p* = 0.000
TMD	Kestane and Bas (2024) [24]	123.29 ± 17.08	106.05 ± 13.67	*p* = 0.010	N/A	N/A	N/A	4.24 ± 0.70	2.38 ± 0.46	*p* = 0.010	N/A	N/A	N/A	N/A	N/A	N/A	N/A	N/A	N/A	N/A	N/A	N/A	N/A	N/A	N/A	N/A
LCMD		117.14 ± 13.33	103.05 ± 10.93	*p* = 0.010	N/A	N/A	N/A	3.85 ± 0.70	2.50 ± 0.55	*p* = 0.010	N/A	N/A	N/A	N/A	N/A	N/A	N/A	N/A	N/A	N/A	N/A	N/A	N/A	N/A	N/A	N/A
LFMD		114.14 ± 13.47	102.71 ± 10.79	*p* = 0.010	N/A	N/A	N/A	4.24 ± 1.00	2.67 ± 0.80	*p* = 0.010	N/A	N/A	N/A	N/A	N/A	N/A	N/A	N/A	N/A	N/A	N/A	N/A	N/A	N/A	N/A	N/A
LFD	George et al. (2022) [22]	6.7 ± 2.0 *	6.8 ± 2.4 *	*p* = 0.82	20.0 ± 12.4	16.4 ± 11.3	*p* < 0.0005	6.5 ± 5.6	5.5 ± 5.5	*p* < 0.0005	N/A	N/A	N/A	1.8 ± 0.9 *	1.6 ± 0.7 *	*p* = 0.410	4.9 ± 1.5 *	4.7 ± 1.4 *	*p* = 0.084	2.9 ± 1.3 *	2.8 ± 1.3 *	*p* = 0.270	1.2 ± 0.3 *	1.2 ± 0.3 *	*p* = 0.570	hs-CRP-B: 3.8 ± 2.7 * PI: 3.5 ± 2.6 * *p* = 0.310
	Properzi et al. (2018) [23]	99.2 ± 17.3	97.0 ± 27.4	*p* = 0.070	14.5 ± 12.2	11.64 ± 9.16	*p* = 0.05	2.76 ± 1.52	2.95 ± 4.32	*p* = 0.040	6.0 ± 0.8	5.8 ± 0.8	*p* = 0.390	144.4 ± 59.3	139.9 ± 0.64	*p* = 0.380	202.2 ± 34.8	199.2 ± 41.2	*p* = 0.270	123.7 ± 30.2	122.6 ± 33.3	*p* = 0.034	49.5 ± 10.4	48.3 ± 9.7	*p* = 0.031	N/A
	Ristic-Medic et al. (2020) [21]	5.20 ± 0.45 *	4.84 ± 0.48 *	*p* = 0.032	17.33 ± 3.76 *	14.38 ± 3.17 *	*p* = 0.006	4.07 (3.45–4.43) ^	2.86 (2.53–3.37) ^	*p* = 0.000	N/A	N/A	N/A	2.40 (1.55–2.69) *^	1.29 (1.20–1.57) *^	*p* = 0.000	6.08 ± 0.69 *	4.81 ± 0.82 *	*p* = 0.000	3.96 ± 0.89 *	3.14 ± 0.85 *	*p* = 0.009	1.22 ± 0.12 *	1.28 ± 0.11 *	*p* = 0.041	hs-CRP-B: 2.10 (0.98–3.20) ^ PI: 0.77 (0.54–1.27) ^ *p* = 0.008
LCD	Badali et al. (2023) [18]	N/A	N/A	N/A	N/A	N/A	N/A	N/A	N/A	N/A	N/A	N/A	N/A	136.23 ± 50.50	118.50 ± 49.50	*p* = 0.013	173.35 ± 26.00	162.35 ± 24.63	*p* = 0.006	103.33 ± 27.16	92.22 ± 24.56	*p* = 0.029	42.30 ± 8.61	41.90 ± 7.64	*p* = 0.636	N/A
	Cai et al. (2019) [20]	5.09 ± 0.91	4.94 ± 1.22	N/A	N/A	N/A	N/A	N/A	N/A	N/A	N/A	N/A	N/A	2.65 ± 1.69	2.40 ± 1.70	N/A	4.88 ± 1.38	4.65 ± 1.36	N/A	2.55 ± 0.79	2.51 ± 0.76	N/A	1.16 ± 0.50	1.18 ± 0.51	N/A	N/A
	Rooholahzadegan et al. (2023) [19]	93.12 ± 9.63	90.64 ± 4.69	*p* = 0.013	N/A	N/A	N/A	N/A	N/A	N/A	5.49 ± 0.49	5.56 ± 0.32	*p* = 0.340	N/A	N/A	N/A	N/A	N/A	N/A	N/A	N/A	N/A	10.56 ± 8.65	9.44 ± 6.70	*p* = 0.152	MCP-1 (pg/mL)-B: 11.64 ± 16.48 PI: 110.01 ± 11.79 *p* = 0.603; LPS (pg/mL)-B: 20.72 ± 2.43 PI: 20.90 ± 2.36 *p* = 0.785
TRF 16/8	Cai et al. (2019) [20]	5.12 ± 0.82	5.05 ± 1.35	N/A	N/A	N/A	N/A	N/A	N/A	N/A	N/A	N/A	N/A	2.90 ± 1.75	2.31 ± 1.75	N/A	4.53 ± 1.53	4.37 ± 1.53	N/A	2.73 ± 0.88	2.71 ± 0.87	N/A	1.16 ± 0.45	1.15 ± 0.45	N/A	N/A
	Kord-Varkaneh et al. (2023) [16]	105.36 ± 16.30	92.90 ± 21.20	N/A	9.16 ± 3.22	11.44 ± 4.65	N/A	2.39 ± 0.93	2.59 ± 1.1	N/A	N/A	N/A	N/A	201.50 ± 35.34	133.27 ± 48.67	N/A	190.04 ± 36.58	157.81 ± 33.59	N/A	104.63 ± 27.26	84.04 ± 26.27	N/A	38.18 ± 5.65	39.81 ± 5.72	N/A	hs-CRP-B: 3.14 ± 1.11 PI: 2.02 ± 0.87 *p*: N/A
ADF	Cai et al. (2019) [20]	5.21 ± 0.86	5.19 ± 0.67	N/A	N/A	N/A	N/A	N/A	N/A	N/A	N/A	N/A	N/A	2.80 ± 1.90	2.12 ± 1.90	N/A	4.87 ± 1.02	4.15 ± 1.06	N/A	2.82 ± 0.85	2.79 ± 0.83	N/A	1.23 ± 0.43	1.17 ± 0.38	N/A	N/A
LOV-D	Garousi et al. (2023) [17]	99.05 ± 8.43	90.62 ± 8.77	*p* < 0.001	22.75 ± 17.70	17.80 ± 17.47	*p* < 0.001	5.59 ± 4.42	3.97 ± 3.75	*p* < 0.001	N/A	N/A	N/A	169.43 ± 82.95	121.43 ± 49.42	*p* = 0.001	189.24 ± 26.93	165.94 ± 26.81	*p* < 0.001	114.16 ± 25.75	92.91 ± 24.33	*p* < 0.001	44.75 ± 8.61	46.32 ± 10.37	*p* = 0.354	N/A
SWL-D	Garousi et al. (2023) [17]	97.00 ± 10.16	95.13 ± 8.01	*p* = 0.184	26.77 ± 24.63	27.59 ± 22.59	*p* = 0.550	6.53 ± 6.46	6.56 ± 5.56	*p* = 0.932	N/A	N/A	N/A	171.84 ± 74.45	181.92 ± 81.82	*p* = 0.434	205.97 ± 44.76	208.15 ± 39.38	*p* = 0.765	123.42 ± 38.48	126.78 ± 30.96	*p* = 0.614	47.91 ± 12.17	46.68 ± 11.96	*p* = 0.635	N/A
DASH	Rooholahzadegan et al. (2023) [19]	93.41 ± 9.63	90.76 ± 5.69	*p* = 0.098	N/A	N/A	N/A	N/A	N/A	N/A	5.30 ± 0.35 `	5.09 ± 0.34 `	*p* = 0.314	N/A	N/A	N/A	N/A	N/A	N/A	N/A	N/A	N/A	7.59 ± 3.67	13.47 ± 29.77	*p* = 0.362	MCP-1 (pg/mL)-B: 110.75 ± 7.56 PI: 100.44 ± 10.69 *p* = 0.002; LPS (pg/mL)-B: 21.66 ± 1.83 PI: 18.91 ± 2.98 *p* < 0.001
	Badali et al. (2023) [18]	N/A	N/A	N/A	N/A	N/A	N/A	N/A	N/A	N/A	N/A	N/A	N/A	178.38 ± 73.04	117.05 ± 42.16	*p* < 0.001	203.85 ± 49.84	164.20 ± 30.21	*p* < 0.001	124.77 ± 32.77	87.68 ± 20.54	*p* < 0.001	48.55 ± 9.70	45.85 ± 9.54	*p* = 0.128	N/A
	Sakkarin Chirapongsathorn et al. (2023) [11]	92.5 ± 10.3	92.6 ± 11.3	N/A	N/A	N/A	N/A	N/A	N/A	N/A	N/A	N/A	N/A	132 ± 61.1	117.5 ± 70.8	N/A	197.8 ± 30.4	198.3 ± 41.01	N/A	128.3 ± 33.1	124.8 ± 43.5	N/A	54.9 ± 13.5	56.1 ± 13.09	N/A	N/A
	Razavi Zade et al. (2016) [15]	93.7 ± 12.5	92.6 ± 11.2	*p* = 0.51	12.2 ± 5.2	8.9 ± 5.0	*p* < 0.001	2.9 ± 1.5	2.1 ± 1.4	*p* < 0.001	N/A	N/A	N/A	164.3 ± 64.5	133.0 ± 53.7	*p* = 0.006	187.4 ± 33.9	173.4 ± 33.0	*p* = 0.04	111.8 ± 28.5	100.8 ± 28.2	*p* = 0.110	42.7 ± 5.8	46.1 ± 6.0	*p* = 0.002	hs-CRP-B:4823.1 ± 3358.9 PI: 3598.4 ± 2752.6 *p* = 0.004
KD	Sakkarin Chirapongsathorn et al. (2023) [11]	82.99 ± 11.12	76.8 ± 11.4	N/A	31.93 ± 2.7	29.4 ± 2.9	N/A	102.44 ± 9.67	93.5 ± 5.5	N/A	34.2 ± 5.8 **	29.3 ± 6.6 **	N/A	84.1 ± 11.9	79.3 ± 11.06	N/A	132.3 ± 12.2	122.9 ± 11.2	N/A	N/A	N/A	N/A	N/A	N/A	N/A	N/A
CR	Asghari et al. (2022) [14]	89.62 ± 14.20	N/A	N/A	31.32 ± 3.31	103.93 ± 10.30	N/A	N/A	N/A	N/A	N/A	N/A	N/A	N/A	N/A	N/A	N/A	N/A	N/A	N/A	N/A	N/A	N/A	N/A	N/A	N/A
ND	Asghari et al. (2022) [14]	86.61 ± 10.70	N/A	N/A	30.41 ± 3.39	101.88 ± 8.18	N/A	N/A	N/A	N/A	N/A	N/A	N/A	N/A	N/A	N/A	N/A	N/A	N/A	N/A	N/A	N/A	N/A	N/A	N/A	N/A
CN	Razavi Zade et al. (2016) [15]	93.8 ± 11.2	91.4 ± 10.5	*p* = 0.180	11.7 ± 7.0	10.6 ± 6.2	*p* = 0.150	2.7 ± 1.7	2.5 ± 1.5	*p* = 0.100	N/A	N/A	N/A	158.1 ± 58.2	158.4 ± 96.7	*p* = 0.970	190.3 ± 29.5	188.4 ± 27.6	*p* = 0.680	114.7 ± 32.3	111.9 ± 32.9	*p* = 0.570	44.0 ± 5.9	44.8 ± 6.3	*p* = 0.450	hs-CRP-B: 4957.0 ± 3421.8 PI: 4637.5 ± 2872.0 *p* = 0.080
	Kord-Varkaneh et al. (2023) [16]	102.82 ± 11.71	105.78 ± 14.16	N/A	10.78 ± 3.78	12.15 ± 5.25	N/A	2.77 ± 1.11	3.15 ± 1.41	N/A	N/A	N/A	N/A	187.6 ± 73.61	199.56 ± 87.43	N/A	172.21 ± 37.99	180.72 ± 49.49	N/A	93.73 ± 31.77	97.45 ± 35.46	N/A	34.82 ± 7.65	33.73 ± 6.70	N/A	hs-CRP-B: 2.72 ± 1.04 PI: 2.75 ± 1.13 *p*: N/A

Data are presented as mean ± SD, or median (25th–75th). B and PI are reported as exact values, otherwise marked ^ and displayed as MD. N/A states where there was no data available. Glucose, Triglycerides, Cholesterol, LDL-C, and HDL-C expressed in mg/dL, otherwise marked by * and presented in mmol/L. Insulin expressed in µU/mL, otherwise marked by ** and presented in pmol/L. HbA1c expressed in % otherwise marked ` and presented in mmol/mol. hs-CRP expressed in ng/mL, otherwise presented in mg/L. MCP-1 and LPS expressed in pg/mL. A *p* value > 0.05 is considered NS. HOMA-IR, Homeostatic Model Assessment of Insulin Resistance; HbA1c, Hemoglobin A1c; HDL-C, High-Density Lipoprotein Cholesterol; LDL-C, Low-Density Lipoprotein Cholesterol; hs-CRP, High-Sensitivity C-Reactive Protein; MCP-1, Monocyte Chemoattractant Protein-1; LPS, Lipopolysaccharide; MD, Mean Difference; SD, Standard Deviation; NS, Not significant; N/A, Not Available; B, Baseline; PI, Post-intervention; AHA, American Heart Association guidelines; ND, Normal diet; CN, Control Normal diet; SWL-D, Standard weight-loss diet; CR, Calorie-restricted diet; LCD, Low-calorie diet; LFD, Low-Fat Diet; KD, Ketogenic diet; DASH, Dietary Approaches to Stop Hypertension diet; LOV-D, Lacto-ovo-vegetarian diet; ADF, Alternate-day fasting; TRF, Time-Restricted Feeding 16/8; FLiO, Fatty Liver in Obesity diet; MED, Mediterranean diet; LCMD, Low carbohydrate Mediterranean diet; LFMD, Low-fat Mediterranean diet; TMD, Typical Mediterranean diet.

**Table 6:** Comparisons of changes in glucose and lipid metabolism outcomes along with the inflammatory status between interventions with dietary model patterns in MASLD.

Change in the Glucose and Lipid Metabolism Outcomes Along with Inflammatory Status Between Interventions with Dietary Model Patterns in MASLD												
Compared Dietary Models	Reference	Glucose	Insulin	HOMA-IR	HbA1c	Triglycerides	Cholest-erol	LDL-C	HDL-C	hs-CRP	MCP-1	LPS
AHA vs. FLiO	Mogna-Pelaez et al. (2024) [13]	NS	NS	NS	NS	NS	NS	NS	NS	N/A	N/A	N/A
MED vs. LFD	George et al. (2022) [22]	*p* = 0.430	*p* = 0.067	*p* = 0.170	N/A	*p* = 0.670	*p* = 0.170	*p* = 0.480	*p* = 0.570	*p* = 0.770	N/A	N/A
	Properzi et al. (2018) [23]	*p* = 0.485	*p* = 0.281	*p* = 0.268	*p* = 0.278	*p* = 0.284	*p* = 0.457	*p* = 0.755	*p* = 0.605	N/A	N/A	N/A
	Ristic-Medic et al. (2020) [21]	*p* = 0.145	*p* = 0.475	*p* = 0.142	N/A	*p* = 0.048	*p* = 0.760	*p* = 0.857	*p* = 0.041	*p* = 0.239	N/A	N/A
TMD vs. LCMD vs. LFMD	Kestane and Bas (2024) [24]	*p* = 0.610	N/A	*p* = 0.320	N/A	N/A	N/A	N/A	N/A	N/A	N/A	N/A
TRF 16/8 vs. CN	Kord-Varkaneh et al. (2023) [16]	*p* = 0.015	*p* = 0.880	*p* = 0.246	N/A	*p* ˂ 0.001	*p* ˂ 0.001	*p* = 0.014	*p* = 0.159	*p* ˂ 0.001	N/A	N/A
ADF vs. CN	Cai et al. (2019) [20]	NS	NS	N/A	N/A	*p* ˂ 0.001	*p* ˂ 0.001	NS	NS	N/A	N/A	N/A
TRF vs. CN		NS	NS	N/A	N/A	*p* ˂ 0.001	NS	NS	NS	N/A	N/A	N/A
DASH vs. LCD	Rooholahzadegan et al. (2023) [19]	*p* = 0.923	N/A	N/A	*p* ˂ 0.001	N/A	N/A	N/A	*p* = 0.080	N/A	*p* = 0.027	*p* = 0.011
	Badali et al. (2023) [18]	N/A	N/A	N/A	N/A	*p* = 0.037	*p* = 0.011	*p* = 0.002	*p* = 0.649	N/A	N/A	N/A
DASH vs. KD	Sakkarin Chirapongsathorn et al. (2023) [11]	*p* = 0.720	N/A	N/A	N/A	*p* = 0.170	*p* = 0.730	*p* = 0.509	*p* = 0.220	N/A	N/A	N/A
DASH vs. CN	Razavi Zade et al. (2016) [15]	*p* = 0.610	*p* = 0.010	*p* = 0.010	N/A	*p* = 0.040	*p* = 0.150	*p* = 0.330	*p* = 0.080	*p* = 0.030	N/A	N/A
LOV-D vs. SWL-D	Garousi et al. (2023) [17]	*p* = 0.001	*p* = 0.006	*p* ˂ 0.001	N/A	*p* = 0.006	*p* ˂ 0.001	*p* ˂ 0.001	*p* = 0.770	N/A	N/A	N/A
CR vs. ND	Asghari et al. (2022) [14]	*p* = 0.140	N/A	N/A	N/A	*p* = 0.110	*p* = 0.030	*p* = 0.115	*p* = 0.890	N/A	N/A	N/A

Data are presented as *p*-values and color-coded along with the level of significance in the change of reported PI values between interventions with model diets. Colors in table refer to significant (green) and not significant (red) change in the reported PI values. N/A states where there was no data available (Yellow shadowing in the table). A *p*-value > 0.05 is considered NS. HOMA-IR, Homeostatic Model Assessment of Insulin Resistance; HbA1c, Hemoglobin A1c; HDL-C, High-Density Lipoprotein Cholesterol; LDL-C, Low-Density Lipoprotein Cholesterol; hs-CRP, High-Sensitivity C-Reactive Protein; NS, Not significant; N/A, Not Available; AHA, American Heart Association guidelines; ND, Normal diet; CN, Control Normal diet; SWL-D, Standard weight-loss diet; CR, Calorie-restricted diet; LCD, Low-calorie diet; LFD, Low-Fat Diet; KD, Ketogenic diet; DASH, Dietary Approaches to Stop Hypertension diet; LOV-D, Lacto-ovo-vegetarian diet; ADF, Alternate-day fasting; TRF, Time-Restricted Feeding 16/8; FLiO, Fatty Liver in Obesity diet; MED, Mediterranean diet; LCMD, Low carbohydrate Mediterranean diet; LFMD, Low-fat Mediterranean diet; TMD, Typical Mediterranean diet.

**Table 7:** Changes in liver function outcomes upon intervention with dietary pattern models of the RCT studies conducted in MASLD patients.

Dietary Pattern Model		Liver Function Outcomes														
		Hepatic Enzymes									Hepatic Steatosis			Liver Fibrosis	Hepatic Inflammation	Grade of Fatty Liver
		AST			ALT			ALP			CAP					
		B	PI	*p*	B	PI	*p*	B	PI	*p*	B	PI	*p*			
AHA	Mogna-Pelaez et al. (2024) [13]	23.63 ± 10.92	0.31 ± 6.53 ^	NS	21.49 ± 14.34	−2.72 ± 15.76 ^	NS	N/A	N/A	N/A	3.62 ± 1.93	−0.12 ± 4.09 ^	NS	LSM-B: 4.26 ± 0.8 PI: 0.57 ± 0.49 ^ *p*: NS; ARFI-B: 1.25 ± 0.32 PI: 0.05 ± 0.14 ^ *p*: NS; IHL-B: 27.26 ± 38.48 PI: −4.72 ± 34.14 ^ *p*: NS	FLI-B: 17.00 ± 18.10 PI: −11.03 ± 21.18 ^ *p* < 0.005	
FLiO diet	Mogna-Pelaez et al. (2024) [13]	23.63 ± 10.92	−1.08 ± 6.84 ^	NS	21.49 ± 14.34	−9.35 ± 15.60 ^	*p* < 0.001	N/A	N/A	N/A	3.62 ± 1.93	−1.09 ± 2.84 ^	NS	LSM-B: 4.26 ± 0.8 PI:-0.90 ± 0.54 ^ *p*: NS; ARFI-B: 1.25 ± 0.32 PI: 0.14 ± 0.14 ^ *p*: NS; IHL-B: 27.26 ± 38.48 PI:- 10.46 ± 18.57 ^ *p*: NS	FLI-B: 17.00 ± 18.10 PI:-21.76 ± 18.92 ^ *p* < 0.001	
MED	George et al. (2022) [22]	31.8 ± 12.6	39.7 ± 27.4	*p* = 0.290	54.1 ± 25.2	64.7 ± 39.5	*p* = 0.032	91.5 ± 27.7	96.8 ± 31.3	*p* = 0.520	13.7 ± 7.8	12.1 ± 7.8	*p* = 0.069	IHL-B: 90.1 ± 74.6 PI: 105.1 ± 91.2 *p* = 0.260	N/A	
	Properzi et al. (2018) [23]	N/A	N/A	N/A	77 ± 51	69 ± 47	*p* = 0.049	N/A	N/A	N/A	34.2 ± 16.3	24.0 ± 14.7	*p* < 0.001	HepaScore-B: 0.39 ± 0.33 PI: 0.41 ± 0.32 *p* = 0.780; IHL-B: 102 ± 120 PI: 83 ± 99 *p* < 0.001	N/A	
	Ristic-Medic et al. (2020) [21]	32.50 (23.00–32.75)	20.00 (16.00–21.75)	*p* = 0.000	65.33 ± 23.90	27.33 ± 6.46	*p* = 0.000	N/A	N/A	N/A	N/A	N/A	N/A	IHL-B: 47.42 ± 36.25 PI: 24.33 ± 11.57 *p* = 0.000	FLI-B: 81.92 ± 9.95 PI: 43.17 ± 7.99 *p* = 0.000; HSI-B: 47.6 ± 4.92 PI: 39.34 ± 3.24 *p* = 0.000	
TMD	Kestane and Bas (2024) [24]	42.19 ± 13.36	26.76 ± 7.08	*p* = 0.010	69.19 ± 14.48	48.52 ± 9.00	*p* = 0.010	N/A	N/A	N/A	N/A	N/A	N/A	FIB-4-B: 0.61 ± 0.22 PI: 0.48 ± 0.16 *p* = 0.010; IHL-B: 35.38 ± 7.05 PI: 20.81 ± 5.73 *p* = 0.010	FLI-B: 85.62 ± 7.34 PI: 60.38 ± 13.15 *p* = 0.010	
LCMD		45.71 ± 14.62	34.90 ± 8.17	*p* = 0.010	66.38 ± 11.36	51.43 ± 6.34	*p* = 0.010	N/A	N/A	N/A	N/A	N/A	N/A	FIB-4-B: 0.73 ± 0.28 PI: 0.61 ± 0.20 *p* = 0.010; IHL-B: 39.33 ± 11.13 PI: 31.24 ± 8.17 *p* = 0.010	FLI-B: 87.90 ± 8.79 PI: 71.95 ± 17.18 *p* = 0.010	
LFMD		50.43 ± 16.50	37.24 ± 10.60	*p* = 0.010	68.67 ± 15.30	51.95 ± 10.79	*p* = 0.010	N/A	N/A	N/A	N/A	N/A	N/A	FIB-4-B: 0.73 ± 0.22 PI: 0.62 ± 0.17 *p* = 0.010; IHL-B: 43.29 ± 12.10 PI: 30.76 ± 8.26 *p* = 0.010	FLI-B: 88.71 ± 4.77 PI: 68.71 ± 10.97 *p* = 0.010	
LFD	George et al. (2022) [22]	35.65 (25.00–41.50)	25.50 (18.75–30.75)	*p* = 0.006	63.17 ± 15.76	31.92 ± 11.89	*p* = 0.000	N/A	N/A	N/A	N/A	N/A	N/A	IHL-B: 42.53 ± 10.48 PI: 27.08 ± 9.90 *p* = 0.000	FLI-B: 83.52 ± 10.76 PI: 55.08 ± 18.22 *p* = 0.000; HSI-B: 45.85 ± 4.63 PI: 37.67 ± 4.07 *p* = 0.000	
	Properzi et al. (2018) [23]	41.8 ± 21.6	34.1 ± 15.4	*p* = 0.040	61.5 ± 37.0	46.9 ± 20.8	*p* = 0.009	93.3 ± 33.3	94.2 ± 24.9	*p* = 0.710	9.2 ± 10.7	8.9 ± 12.4	*p* = 0.020	IHL-B: 126.7 ± 128.8 PI: 95.2 ± 72.9 *p* = 0.029	N/A	
	Ristic-Medic et al. (2020) [21]	N/A	N/A	N/A	68 ± 66	56 ± 45	*p* = 0.004	N/A	N/A	N/A	21.5 ± 10.0	15.3 ± 7.7	*p* < 0.001	HepaScore-B: 0.26 ± 0.28 PI: 0.30 ± 0.29 *p* = 0.090; IHL-B: 121 ± 125 PI: 102 ± 110 *p* = 0.055	N/A	
LCD	Badali et al. (2023) [18]	26.75 ± 9.28	25.05 ± 8.70	*p* = 0.067	37.35 ± 18.37	31.60 ± 16.24	*p* = 0.019	N/A	N/A	N/A	N/A	N/A	N/A	N/A	N/A	
	Cai et al. (2019) [20]	26.75 ± 9.28	25.05 ± 8.70	*p* = 0.067	37.35 ± 18.37	31.60 ± 16.24	*p* = 0.019	N/A	N/A	N/A	N/A	N/A	N/A	N/A	N/A	
	Rooholahzadegan et al. (2023) [19]	N/A	N/A	N/A	N/A	N/A	N/A	N/A	N/A	N/A	N/A	N/A	N/A	N/A	N/A	
TRF 16/8	Cai et al. (2019) [20]	N/A	N/A	N/A	N/A	N/A	N/A	N/A	N/A	N/A	N/A	N/A	N/A		N/A	
	Kord-Varkaneh et al. (2023) [16]	26.31 ± 6.16	20.50 ± 4	N/A	34.04 ± 13.88	21.22 ± 5.38	N/A	N/A	N/A	N/A	N/A	N/A	N/A	LSM-B: 6.33 ± 1.01 PI: 5.15 ± 1.15 *p*: N/A; IHL-B: 33.00 ± 14.99 PI: 23.18 ± 11.05 *p*: N/A	N/A	
ADF	Cai et al. (2019) [20]	N/A	N/A	N/A	N/A	N/A	N/A	N/A	N/A	N/A	N/A	N/A	N/A	N/A	N/A	
LOV-D	Garousi et al. (2023) [17]	30.94 ± 13.20	22.43 ± 6.93	*p* < 0.001	46.02 ± 28.58	24.70 ± 15.82	*p* < 0.001	N/A	N/A	N/A	N/A	N/A	N/A	N/A	N/A	
SWL-D	Garousi et al. (2023) [17]	29.94 ± 11.14	25.78 ± 9.36	*p* = 0.013	42.26 ± 23.37	32.10 ± 18.55	*p* = 0.004	N/A	N/A	N/A	N/A	N/A	N/A	N/A	N/A	
DASH	Rooholahzadegan et al. (2023) [19]	24.10 ± 10.91	18.40 ± 6.57	*p* = 0.005	27.20 ± 14.0	18.40 ± 6.57	*p* = 0.001	N/A	N/A	N/A	N/A	N/A	N/A	N/A	N/A	
	Badali et al. (2023) [18]	24.10 ± 10.91	18.40 ± 6.57	*p* = 0.005	24.3 ± 10.3	18.75 ± 8.91	*p* = 0.001	N/A	N/A	N/A	N/A	N/A	N/A		N/A	
	Sakkarin Chirapongsathorn et al. (2023) [11]	19.8 ± 6.1	20 ± 4.5	N/A	28.6 ± 12.1	23.1 ± 8.7	N/A	N/A	N/A	N/A	N/A	N/A	N/A	LSM-B: 5.01 ± 1.2 PI: 4.3 ± 0.7 *p*: N/A	N/A	
	Razavi Zade et al. (2016) [15]	42.7 ± 34.1	32.0 ± 16.6	*p* = 0.020	36.4 ± 19.1	28.0 ± 20.8	*p* = 0.010	206.2 ± 54.8	179.9 ± 55.9	*p* < 0.001	N/A	N/A	N/A		N/A	% B: Grade I 6 ± 20.0; Grade II 14 ± 46.7; Grade III 10 ± 33.3; % PI: Grade I 20 ± 66.7; Grade II 10 ± 33.3; Grade III 0 ± 0.0
KD	Sakkarin Chirapongsathorn et al. (2023) [11]	29.2 ± 11.6	22.6 ± 4.8	N/A	44.08 ± 21.3	28.6 ± 12.1	N/A	N/A	N/A	N/A	N/A	N/A	N/A	LSM-B: 5.9 ± 3.1 PI: 5.2 ± 1.7 *p*: N/A	N/A	
CR	Asghari et al. (2022) [14]	33.66 ± 12.62	29.58 ± 12.57	*p* = 0.160	43.58 ± 26.38	39.25 ± 24.21	*p* = 0.040	N/A	N/A	N/A	N/A	N/A	N/A	N/A	N/A	(n) B: Grade 0-0, Grade I-10, Grade II -17, Grade III-3; (n) PI: Grade 0-0, Grade I-11, Grade II -17, Grade III-2.
ND	Asghari et al. (2022) [14]	29.85 ± 9.80	34.27 ± 21.06	*p* = 0.040	33.71 ± 20.36	40.94 ± 28.81	*p* = 0.070	N/A	N/A	N/A	N/A	N/A	N/A	N/A	N/A	
CN	Razavi Zade et al. (2016) [15]	N/A	N/A	N/A	N/A	N/A	N/A	N/A	N/A	N/A	N/A	N/A	N/A		N/A	% B: Grade I 8 ± 26.7; Grade II 14 ± 46.7; Grade III 8 ± 26.7; % PI: Grade I 19 ± 63.3; Grade II 5 ± 16.7; Grade III 6 ± 20.0
	Kord-Varkaneh et al. (2023) [16]	23.68 ± 8.20	23.77 ± 9.66	N/A	30.34 ± 5.13	28.04 ± 8.12	N/A	N/A	N/A	N/A	N/A	N/A	N/A	LSM-B: 5.82 ± 1.44 PI: 5.46 ± 1.32 *p*: N/A; IHL-B: 34.77 ± 12.93 PI: 39.22 ± 36.95 *p*: N/A	N/A	

Data are presented as mean ± SD, or median (25th–75th). B and PI are reported as exact values, otherwise marked ^ and displayed as MD. N/A states where there was no data available. AST and ALT expressed in IU/L. IHL expressed in %. CAP expressed in dB/m. ARFI expressed in m/s. LSM expressed in kPa. FLI and HSI Index, and FIB-4 score are presented as exact values. A *p*-value > 0.05 is considered NS. AST, Aspartate aminotransferase; ALT, Alanine aminotransferase; IHL, Intrahepatic lipid; CAP, Controlled Attenuation Parameter; LSM, Liver stiffness measurement; ARFI, Acoustic Radiation Force Impulse elastography; FLI Index, Fatty Liver Index; HSI Index, Hepatic steatosis index; FIB-4, Fibrosis-4 score. MD, Mean Difference; SD, Standard Deviation; NS, Not significant; N/A, Not Available; B, Baseline; PI, Post-intervention; AHA, American Heart Association guidelines; ND, Normal diet; CN, Control Normal diet; SWL-D, Standard weight-loss diet; CR, Calorie-restricted diet; LCD, Low-calorie diet; LFD, Low-Fat Diet; KD, Ketogenic diet; DASH, Dietary Approaches to Stop Hypertension diet; LOV-D, Lacto-ovo-vegetarian diet; ADF, Alternate-day fasting; TRF, Time-Restricted Feeding 16/8; FLiO, Fatty Liver in Obesity diet; MED, Mediterranean diet; LCMD, Low carbohydrate Mediterranean diet; LFMD, Low-fat Mediterranean diet; TMD, Typical Mediterranean diet.

**Table 8:** Comparisons of changes in liver function outcomes upon intervention with dietary pattern models of the RCT studies conducted in MASLD patients.

Change in the Liver Function Outcomes Between Interventions with Dietary Model Patterns in MASLD													
Compared Dietary Models	Reference	AST	ALT	GGT	IHL	CAP	LSM	ARFI	FLI Index	HSI Index	Hepascore	FIB-4 Score	Liver Pathology Staging
AHA vs. FLiO	Mogna-Pelaez et al. (2024) [13]	NS	NS	NS	NS	NS	NS	NS	*p* = 0.054	N/A	N/A	N/A	N/A
MED vs. LFD	George et al. (2022) [22]	*p* = 0.073	*p* = 0.017	*p* = 0.042	*p* = 0.870	N/A	*p* = 0.580	N/A	N/A	N/A	N/A	N/A	N/A
	Properzi et al. (2018) [23]	N/A	*p* = 0.363	*p* = 0.716	*p*= 0.722	N/A	*p* = 0.697	N/A	N/A	N/A	*p* = 0.885	N/A	N/A
	Ristic-Medic et al. (2020) [21]	*p* = 0.017	*p* = 0.128	*p* = 0.224	N/A	N/A	N/A	N/A	*p* = 0.021	*p* = 0.435	N/A	N/A	N/A
TMD vs. LCMD vs. LFMD	Kestane and Bas (2024) [24]	*p* = 0.010	*p* = 0.410	*p* = 0.010	N/A	N/A	N/A	N/A	*p* = 0.003	N/A	N/A	*p* = 0.020	N/A
TRF 16/8 vs. CN	Kord-Varkaneh et al. (2023) [16]	*p* = 0.010	*p* = 0.013	*p* = 0.026	N/A	*p* = 0.009	*p* ˂ 0.001	N/A	N/A	N/A	N/A	N/A	N/A
ADF vs. CN	Cai et al. (2019) [20]	N/A	N/A	N/A	N/A	N/A	NS	N/A	N/A	N/A	N/A	N/A	N/A
TRF vs. CN		N/A	N/A	N/A	N/A	N/A	NS	N/A	N/A	N/A	N/A	N/A	N/A
DASH vs. LCD	Rooholahzadegan et al. (2023) [19]	*p* = 0.008	*p* = 0.149	N/A	N/A	N/A	N/A	N/A	N/A	N/A	N/A	N/A	N/A
	Badali et al. (2023) [18]	*p* = 0.202	*p* = 0.240	N/A	N/A	*p* = 0.590	*p* = 0.120	N/A	N/A	N/A	N/A	N/A	N/A
DASH vs. KD	Sakkarin Chirapongsathorn et al. (2023) [11]	*p* = 0.040	*p* = 0.053	N/A	N/A	*p* = 0.053	*p* = 0.970	N/A	N/A	N/A	N/A	N/A	N/A
DASH vs. CN	Razavi Zade et al. (2016) [15]	*p* = 0.060	*p* = 0.020	N/A	N/A	N/A	N/A	N/A	N/A	N/A	N/A	N/A	*p* = 0.003
LOV-D vs. SWL-D	Garousi et al. (2023) [17]	*p* = 0.08	*p* = 0.04	N/A	N/A	N/A	N/A	N/A	N/A	N/A	N/A	N/A	N/A
CR vs. ND	Asghari et al. (2022) [14]	*p* = 0.020	*p* = 0.010	N/A	N/A	N/A	N/A	N/A	N/A	N/A	N/A	N/A	*p* = 0.350

Data are presented as *p*-values and color-coded along with the level of significance in the change of reported PI values between interventions with model diets. Colors in table refer to significant (green) and not significant (red) change in the reported PI values. N/A states where there was no data available (Yellow shadowing in the table). AST, ALT, and GGT expressed in IU/L. A *p*-value > 0.05 is considered NS. AST, Aspartate aminotransferase; ALT, Alanine aminotransferase; GGT, Gamma-glutamyl transpeptidase; IHL, Intrahepatic lipid; CAP, Controlled Attenuation Parameter; LSM, Liver stiffness measurement; ARFI, Acoustic Radiation Force Impulse elastography; FLI Index, Fatty Liver Index; HSI Index, Hepatic steatosis index; FIB-4, Fibrosis-4 score. NS, Not significant; N/A, Not Available; AHA, American Heart Association guidelines; ND, Normal diet; CN, Control Normal diet; SWL-D, Standard weight-loss diet; CR, Calorie-restricted diet; LCD, Low-calorie diet; LFD, Low-Fat Diet; KD, Ketogenic diet; DASH, Dietary Approaches to Stop Hypertension diet; LOV-D, Lacto-ovo-vegetarian diet; ADF, Alternate-day fasting; TRF, Time-Restricted Feeding 16/8; FLiO, Fatty Liver in Obesity diet; MED, Mediterranean diet; LCMD, Low carbohydrate Mediterranean diet; LFMD, Low-fat Mediterranean diet; TMD, Typical Mediterranean diet.
